# Acrylonitrile adducts: design, synthesis and biological evaluation as antimicrobial, haemolytic and thrombolytic agent

**DOI:** 10.1038/s41598-023-33605-1

**Published:** 2023-04-17

**Authors:** Parineeta Das, Nirmala Devi, Nisha Gaur, Swagata Goswami, Dhiraj Dutta, Rama Dubey, Amrit Puzari

**Affiliations:** 1 Department of Chemistry, National Institute of Technology Nagaland, Chumoukedima, Nagaland 797103 India; 2 Chulalongkorn University, Bangkok, 10330 Thailand; 3 Defence Research Laboratory, Post Bag No. 2, Tezpur, Assam 784001 India

**Keywords:** Biochemistry, Microbiology, Environmental sciences

## Abstract

In this work, five acrylonitrile adducts were screened for antibacterial activity against Gram-positive *Bacillus subtilis*, Microbial Type Culture Collection and Gene Bank (MTCC 1305) and Gram-negative *Escherichia coli* (MTCC 443). Synthesis was followed by aza-Michael addition reaction, where the acrylonitrile accepts an electron pair from the respective amines and results in the formation of n-alkyliminobis-propionitrile and n-alkyliminopropionitrile under microwave irradiation. Characterization of the compounds were performed using Fourier Transform Infrared (FTIR), Proton Nuclear Magnetic Resonance (^1^H NMR) and Electrospray Ionisation Mass Spectrometry (ESI–MS). The particle size characterization was done by Dynamic Light Scattering (DLS) technique. The antibacterial study showed higher inhibition rate for both Gram-positive and Gram-negative bacteria. The antibacterial ability was found to be dose dependent. The minimum inhibitory concentration against both bacteria were found to be 1, 3, 0.4, 1, 3 µl/ml for *E. coli* and 6, 6, 0.9, 0.5, 5 µl/ml for *B. subtilis*. Time-kill kinetics evaluation showed that the adducts possess bacteriostatic action. Further it was evaluated for high-throughput in vitro assays to determine the compatibility of the adducts for drug delivery. The haemolytic and thrombolytic activity was analysed against normal mouse erythrocytes. The haemolytic activity showed prominent results, and thereby projecting this acrylonitrile adducts as potent antimicrobial and haemolytic agent.

## Introduction

In recent years, the majority of infectious diseases have been linked to bacteria, as a result of widespread antibiotic resistance among bacteria strains. According to the 2016 United Nations (UN) declaration on antimicrobial resistance^1,2^, global plans and initiatives to address antimicrobial resistance are advancing. If current trends continue, bacterial infections with multidrug-resistant traits will be listed among the top three global public health risks, causing up to 10 million fatalities worldwide by 2050^3–5^. Antimicrobial resistance against accessible medications has evolved from the availability of poor treatment and widespread usage of antimicrobial agents. There are various reports on studies of nanocomposites and nanoparticles as effective antimicrobial agent. Hasanin et al. has reported several works on doping of nanocomposites and showed that doping a material with the nanocomposite enhances the antimicrobial activity against various gram positive and gram negative bacteria^6^. They have proved that silver nanoparticles (Ag-NPs) loaded nanocomposites has high potential as antimicrobial agent^7^. Loading of the nanocomposites was carried out through ecofriendly method^8,9^ and incorporation of the nanocomposites enhances the antimicrobial, antifungal, antiviral, antioxidant and anticancer activities^10,11^. Samuel et al*.*^12^ developed simple one-pot method and promoted the biological synthesis of nanoparticles as a promising method for nanoparticle synthesis. They have developed a hybrid polymer composite of lithocholic acid (LCA), zinc pyrithione (Zn) and cinnamaldehyde (Cn) (named as LCA–Zn–Cn) which displays potent antibacterial/antibiofilm activity^13^. Moreover, they have biosynthesized iron oxide nanoparticle (Fe_3_O_4_ NPs) and showed its dual benefits as effective adsorbent for removal of heavy metal ion from wastewater and as biomedical agents^12^.

Morita–Baylis–Hillman adducts (MBHA) derived from isatin derivatives and acrylonitrile has showed bactericidal and fungicidal properties by preventing the growth of the majority of microorganism species utilized in biological experiments^14^. The adduct 2-(3-hydroxy-2-oxoindolin-3-yl)acrylonitrile (ISACN) has shown as a promising anti-inflammatory agent^15^. MBHA obtained using acrylonitrile as Michael acceptor inhibited the growth of various species of bacteria and fungi showing minimum inhibitory concentration of 32 µg/ml which reflects the antimicrobial effectiveness of acrylonitrile adducts^14^. The acyl derivatives enantiomers of the Morita–Baylis–Hillman adduct (±)-2-[Hydroxy(*m*-nitrophenyl)methyl]acrylonitrile and (±)-2-[Hydroxy(*p*-nitrophenyl)methyl]acrylonitrile showed no toxicity in peritoneal macrophages from Swiss mice^16^. Rocha et al.^17^ has reported that MBHA exhibits no haemolytic activity to human erythrocytes. These adducts can serve as a potential drug candidate for treating various diseases.

Investigation of aza-Michael adducts in the field of biological studies has not been explored much. Thus the noticeable lack of novel compounds as effective antimicrobials to suppress the microbial growth encouraged us to evaluate the acrylonitrile adducts as efficient antimicrobial agents. The use of nitrogen based molecules in the field of pharmaceuticals is expanding day-by-day^18^. The major-league in the structural diversity of *N*-heterocyclic compounds made it recognizable as an effective molecule in the search of new drugs^19^. Large numbers of clinically approved drugs have developed multidrug resistance leading to adverse side effects. In medicinal chemistry, investigation on nitrogen-based molecules has progressively become an attractive area of research. Study on the antibacterial activity of the alkaloids shows the efficacy of the nitrogen constituent in the molecule^20^. Metronidazole and quinolones are prominent antibacterial drugs derived from naturally occurring alkaloids^21^. Thus, many pharmaceutical significant drug molecules contain ‘Nitrogen’ as a constituent in the molecule. Bioactive nitrogen containing components shows potent antimicrobial activity against gram-positive and gram-negative bacteria^22–24^. Advantage of having a nitrogen atom and proton-donating amine hydrogen atoms in the molecule is that it facilitates the formation of hydrogen bonds with enzymes, proteins and receptors^21^. On a similar note organic molecules containing –C≡N as the functional group, such as nitriles, constitutes a series of diverse organic molecules with diverse structures^25–29^. The medical world uses a variety of nitrile-containing drugs with various structures^30–32^. Medicinal compounds with unsaturated –C≡N can be conjugated either with additional electron withdrawing groups or heteroatoms. Egelkamp et al*.*^33^ reported effects of various nitrile containing compounds on bacterial communities. They made a comparative study between the nitrile compounds and their corresponding degradation products. The study revealed distinct impact of nitrile groups on the bacterial communities compared to the compounds with carboxylic acid groups. Therefore, there is sufficient scope to investigate the efficacy of nitrile compounds as antibacterial agent^34^.

Aza-Michael addition reaction can be considered as a simple as well as convenient synthetic route for synthesis of novel compounds through C–N bond formation. The reaction between several primary aliphatic amines and acrylonitrile has been carried out in our previous work^35^. We have developed a series of aza-Michael adduct which has potent antimicrobial activity and shows no haemolysis. One-pot synthesis, short reaction time, cost-effectiveness, easy availability, antimicrobial and biological assay of acrylonitrile adducts marks the novelty of the work. These adducts have multiple applications. It has been reported that the adducts showed prominent adsorption of Fe^2+^ ion from aqueous media^36^. In addition to it, this work aims to investigate the activity of acrylonitrile adducts against antimicrobial and biological properties. Till date, very few reports are available on the application of acrylonitrile adducts as potent antimicrobial agent which prompted us to undertake this study with a series of aza-Michael adducts to evaluate the efficiency of such molecules specifically for bio-medical applications. The aza-Michael adducts used for the study were synthesized under microwave irradiation using literature procedure^35^ under mild conditions. Furthermore, the five acrylonitrile adducts were also investigated for biological studies. The breakdown of red blood cells caused by the rupture of the lipid bilayer of the cell membrane and releasing of intracellular contents is called haemolysis. Because of easy availability, isolation and having similar properties to those of human cells, the in vitro analysis with the erythrocyte of mouse blood is a beneficial tool^37,38^ for assessing the efficacy of the synthesized compounds for preclinical trials^39^. This study directly indicates the toxicity of injectable drugs. Haemolytic activity was evaluated for acrylonitrile adducts and interaction of the compounds with erythrocyte of mouse blood cell was recorded. We have also carried out the investigation on thrombolytic behaviour^40,41^. As the compounds synthesized from aza-Michael addition reaction are proposed to be used as intermediates for drugs having properties like anti-inflammatory, antibiotics, antimicrobial etc., therefore the blood clot dissolving activity was evaluated for all the synthesized acrylonitrile adducts^42^.

## Materials and methods

### Materials

All the chemicals used for the synthesis of mono and di-adducts of acrylonitrile were procured from Sigma-Aldrich and Tokyo Chemical Industry (TCI) Chemicals, Japan. Molecular sieves of 4 Å beads, 4–8 mesh were purchased from Sigma-Aldrich. Merck silica gel 60 F_254_ plates were used for thin layer chromatography. All the chemical reagents were of analytical grade and were used without further purification. Solvents used during the synthesis were dried by using standard procedure. Mixing of the substrates was carried out by Light-Emitting Diode (LED) Digital Vortex Mixer.

### Antimicrobial activity

The compounds were evaluated for their antimicrobial activities against two bacteria viz. Gram-positive bacterial strain *Bacillus subtilis* (MTCC 1305) and Gram-negative bacterial strain *Escherichia coli* (MTCC 443). Both of the strains were procured from Microbial Type Culture and Collection (MTCC), Chandigarh, India. Maintaining a temperature of 37 °C lyophilized culture was revived in nutrient broth medium by keeping it in a rotatory shaker for 12 h. Using 0.5 McFarland standards each strain was adjusted to a concentration of 10^8^ cells/ml^43–45^.

#### Agar well diffusion assay

The fundamental evaluation for antimicrobial activity of all the compounds was done by agar well diffusion assay. Bacterial cell cultures were inoculated in nutrient broth and kept for approximately 24 h in incubator shaker to attain the concentration of 10^8^ cells/ml. 50 µl of each bacterial culture was uniformly spread on the Nutrient agar (NA) plates after solidification. Once the bacterial culture on each plate was settled down, 100 µl of 1 g/10 ml ceftriaxone (Positive control) and 100 µl of water (negative control) was loaded in the well as a reference. 100 µl of different concentration (2, 4, 6 and 8%) of acrylonitrile adducts were loaded on bacterial plates and then these plates were incubated at 37 °C for 12 h. The diameters of zone of inhibition in each plate were recorded in millimetre (mm)^43,44^.

#### Antibacterial test by dynamic contact

5 ml of the stock culture (Colony Forming Unit, CFU ~ 10^8^) cells/ml was pellet down at 8000 rpm for 15 min. The supernatant layer was discarded, and the remaining broth was removed by washing the pellet thrice. To make up the stock volume, the pellet of each bacteria was suspended with 5 ml of distilled water. Additionally, 10 µl of the bacterial culture was added in to 2 ml of acrylonitrile adducts solution of different concentrations (2%, 4%, 6% and 8%) in 15 ml test tube. The parameters of temperature 37 °C, time 24 h at 120 rpm was taken for incubation of the test tubes in shaker incubator. The presence of viable bacterial cells was measured using plate count method^46,47^. The effect of concentration of acrylonitrile adducts on bacteria removal efficiency was calculated by percentage reduction as follows:

1 $$\text{Percentage reduction}= \frac{(A-B)}{A}\times 100,$$

where A = number of viable bacteria before treatment, B = number of viable bacteria after treatment.

While the log reduction was calculated as follows:

2 $$Log\, reduction={\text{log}}_{10}A- {\text{log}}_{10}B.$$

### Biological studies

#### Haemolytic activity

The synthesized acrylonitrile adducts were analysed for haemolytic activity against normal mouse erythrocytes as per International Organization for Standardisation (ISO) 10993-4 standards. In the context of this research, the mixture of two compounds, mouse blood (3 ml) and 1× Phosphate-Buffered Saline (PBS) (5 ml) was centrifuged at 3000 rpm for 5 min. The pellets were suspended in 5 ml of PBS, and then the sample solution (20 µl) in dimethyl sulphoxide (DMSO) was added to 180 µl of this combination. The mixture was incubated for 30 min at 37 °C, then centrifuged for 5 min at 13,000 rpm and refrigerated. A spectrophotometer was used to detect the free haemoglobin in the supernatant at 576 nm. The negative and positive controls were DMSO and 1% Triton X-100, respectively. The percentage haemolytic activity was calculated using Eq. (3)^39^.

3 $$\text{\%}Hemolysis=\frac{({A}_{t}-{A}_{n})}{{A}_{s}}\times 100,$$

where A_t_ = absorbance of test sample, A_n_ = absorbance of negative control, A_s_ = absorbance of standard (positive control)

#### Thrombolytic investigation

The thrombolytic investigation was conducted out using the approach described in Ref.^39^. Initially, the empty weight of a sterile Eppendorf tube was measured and to allow clot formation, 1 ml of mouse blood was added to it and the content was incubated for 45 min at 37 °C. After the clot had formed, the serum was withdrawn, and the weight of the tube was measured again to calculate the clot weight (y = weight of clot tube − weight of empty tube). A 100 µl of test solution in 10 ml of DMSO was added to the clot, which was then incubated at 37 °C for 180 min. The liquid portion of the tube was discarded after incubation, and the weight of the tube (z) was measured again to quantify the difference in weight before and after thrombolysis, which was expressed as %thrombolysis. DMSO and streptokinase (SK), were taken as the negative and positive control respectively. The % Thromobolysis was calculated as per Eq. (4)^39^.

4 $$\text{\%}Thrombolysis=\frac{z}{y}\times 100,$$

where z = weight after clot lysis, y = weight of clot before lysis.

### Characterization of acrylonitrile adducts

The synthesized acrylonitrile adducts were characterized by FTIR, ^1^H & ^13^C{^1^H}NMR and ESI-Mass spectroscopic techniques. FTIR spectra were recorded in a Cary 630 FTIR equipment in the range between 400 and 4000 cm^−1^. ^1^H NMR spectra were recorded on Bruker Avlll-300 MHz, Switzerland NMR Spectrometer using Tetramethylsilane (TMS) as internal standard and the δ values are reported in ppm. Mass spectral data were recorded on Waters Ultra-Performed Liquid Chromatography-Triple Quadrupole Mass Spectrometry (UPLC-TQD) (ESI–MS) mass spectrometer.

## General method for synthesis of acrylonitrile adducts

**Figure 1:**
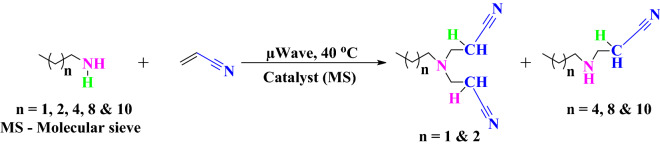
Synthetic route for acrylonitrile adducts.

n-Alkyliminobis-propionitrile and n-alkyliminopropionitrile was synthesized by aza-Michael addition reaction under microwave irradiation using literature procedure^35^ as shown in Fig. 1. In this reaction, 2.5 mmol acrylonitrile was mixed with 1 mmol n-alkylamine in a test tube and 0.12 g of finely powdered molecular sieves (4 Å), which act as a catalyst, was added to the same. After proper mixing, it was subjected to microwave irradiation at 40 °C for a period of 2 h. After the reaction is completed, the content in the test tubes were cooled down to room temperature and the catalyst was removed by centrifugation at a speed of 2500 rpm for 5 min. The supernatant obtained was collected and concentrated under vacuum evaporation.

## Results and discussion

### Characterization

**Figure 2:**
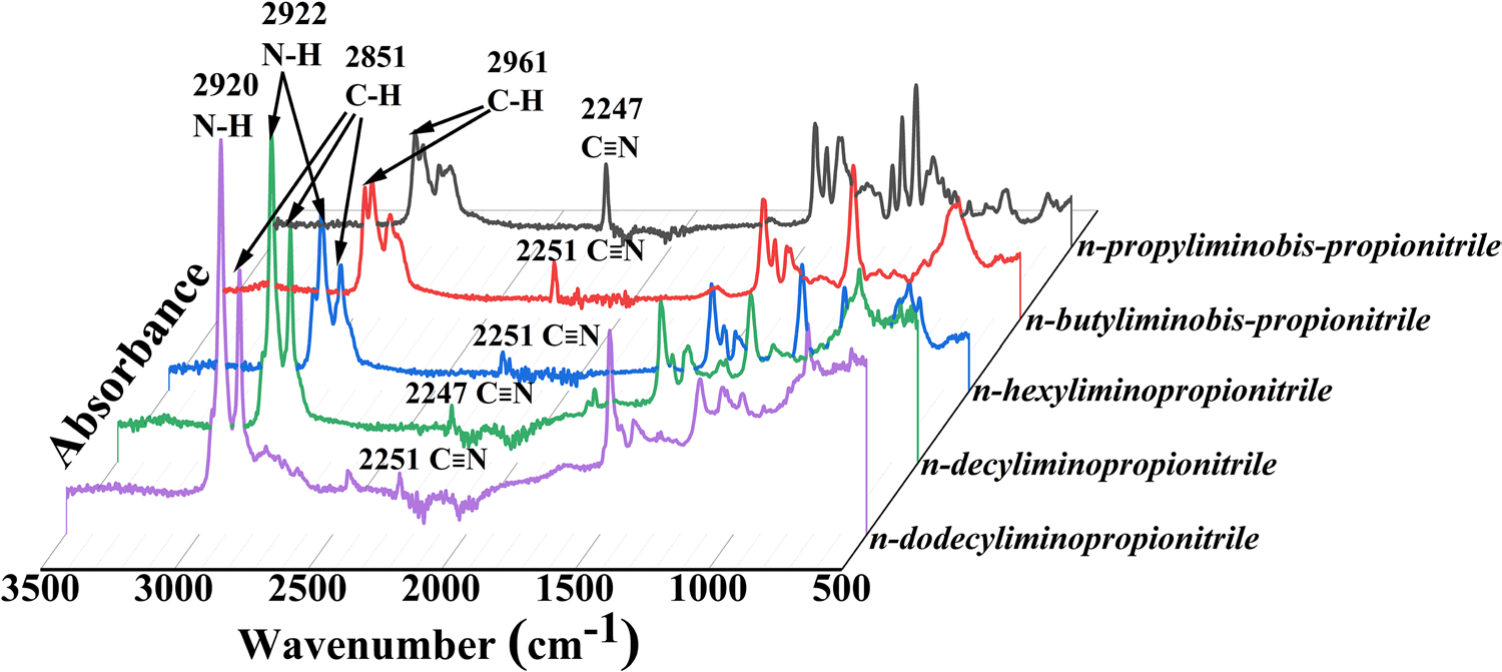
FTIR spectra of acrylonitrile adducts.

FTIR spectra of n-propyliminobis-propionitrile (N1), n-butyliminobis-propionitrile (N2), n-hexyliminopropionitrile (N3), n-decyliminopropionitrile (N4) and n-dodecyliminopropionitrile (N5) are shown in Fig. 2. Appearance of FTIR peaks in the range 2200–2253 cm^−1^ can be attributed to stretching due to –C≡N bond based on available literature^48–51^. The conjugate addition of nitrogen nucleophile, n-propylamine with the Michael acceptor i.e. acrylonitrile leads to the formation of n-alkyliminobis-propionitrile and n-alkyliminopropionitrile. Thiyagarajan et al*.* employed several nucleophiles with acrylonitrile to yield the corresponding Michael adducts^52^. The successful occurrence of aza-Michael addition reaction of acrylonitrile with n-alkylamines was confirmed by the presence of the –C≡N stretching at 2247 and 2251 cm^−1^, which resulted in the formation of alkyliminobis-propionitrile and n-alkyliminopropionitrile. Nitrile bearing compounds are known to possess better inhibitory power against gram positive and gram negative bacteria. The –C≡N stretch for alkyliminobis-propionitrile and n-alkyliminopropionitrile are shown in the Fig. 2.

**Figure 3:**
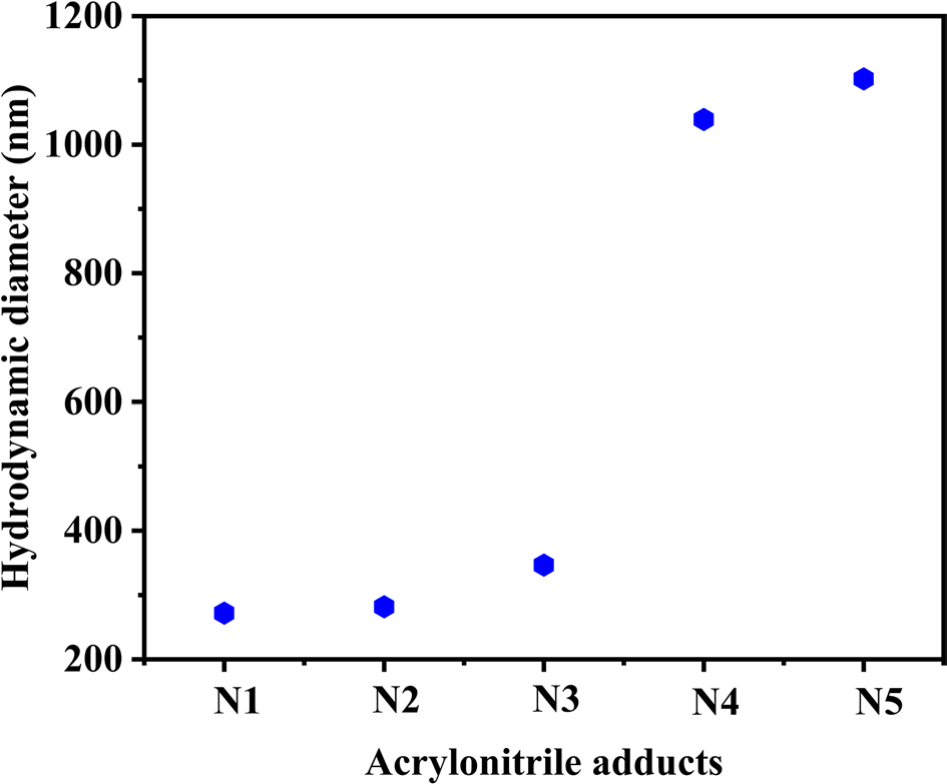
Hydrodynamic diameter of (i) n-propyliminobis-propionitrile **(N1)**, (ii) n-butyliminobis-propionitrile **(N2)**, (iii) n-hexyliminopropionitrile **(N3)**, (iv) n-decyliminopropionitrile **(N4)** and (v) n-dodecyliminopropionitrile **(N5)**.

Nuclear Magnetic Resonance (NMR) studies provided further convincing evidence for the synthesis of all the five acrylonitrile adducts. ^1^H NMR characterization highlights the presence of different functionality in the resulting adducts. Guo et al. carried out Michael addition reaction between diethylamine and acrylic acid 2-phenylsulfanyl-ethyl ester to form 3-Diethylamino-propionic acid 2-phenylsulfanyl-ethyl ester^53^. From the spectroscopic data they revealed that the ^1^H NMR signal for the alkyl group protons attached to the α position of tertiary nitrogen atom appears in the range δ = 3.12–3.74 ppm. On evaluating the ^1^H NMR spectra for five acrylonitrile adducts, it was observed that ^1^H NMR shift for the CH_2_ group attached to α position of tertiary nitrogen appeared at chemical shift value ranging from δ = 2.46–2.9 ppm and the same for the CH_2_ group attached to β position appeared at δ = 1.44 ppm. Results of analysis from ^1^H NMR spectra of n-alkyliminopropionitrile also confirm the presence of α-CH_2_ groups attached to the nitrile group, from similar reasoning. ^1^H NMR peak for this group was observed in the range δ = 2.73–2.92 ppm and the corresponding peak for β-CH_2_ group was observed at δ = 2.99 ppm. ^1^H NMR peak for the terminal methyl protons attached to alkyl chain was observed in the range δ = 0.88–0.90 ppm. Proton NMR spectra for all the five acrylonitrile adducts (N1 to N5) were found identical with the ones reported in literature^35^ (Fig. S2) and thereby confirming the formation of the adducts. Further evidence in support of the formation of the acrylonitrile adducts were obtained from mass spectroscopic data. ESI–MS of n-propyliminobis-propionitrile (N1), n-butyliminobis-propionitrile (N2), n-hexyliminopropionitrile (N3), n-decyliminopropionitrile (N4) and n-dodecyliminopropionitrile (N5) displayed peak at m/z value 164, 180, 155, 211 and 239 respectively, which corresponds with the molecular weight of cationic species [M+1]^+^ i.e. [C_9_H_15_N_3_]^**+**^, [C_10_H_17_N_3_]^+^, [C_9_H_18_N_2_]^+^, [C_13_H_26_N_2_]^+^, [C_15_H_30_N_2_]^+^ (Fig. S3). The particle size was analysed using dynamic light scattering (DLS) technique. During the analysis the particle size was found to appear larger, which is because of the accumulation of water molecule. Average size of n-propyliminobis-propionitrile (N1), n-butyliminobis-propionitrile (N2) and n-hexyliminopropionitrile (N3) are in the range of 250–350 nm. This type of nitrogen containing nanostructures are found in a wide range of pharmaceutical components which are used as building blocks in the process of synthesizing biologically active molecules^54^. Figure 3 shows the hydrodynamic diameter of five acrylonitrile adducts.

### Antibacterial test

**Figure 4:**
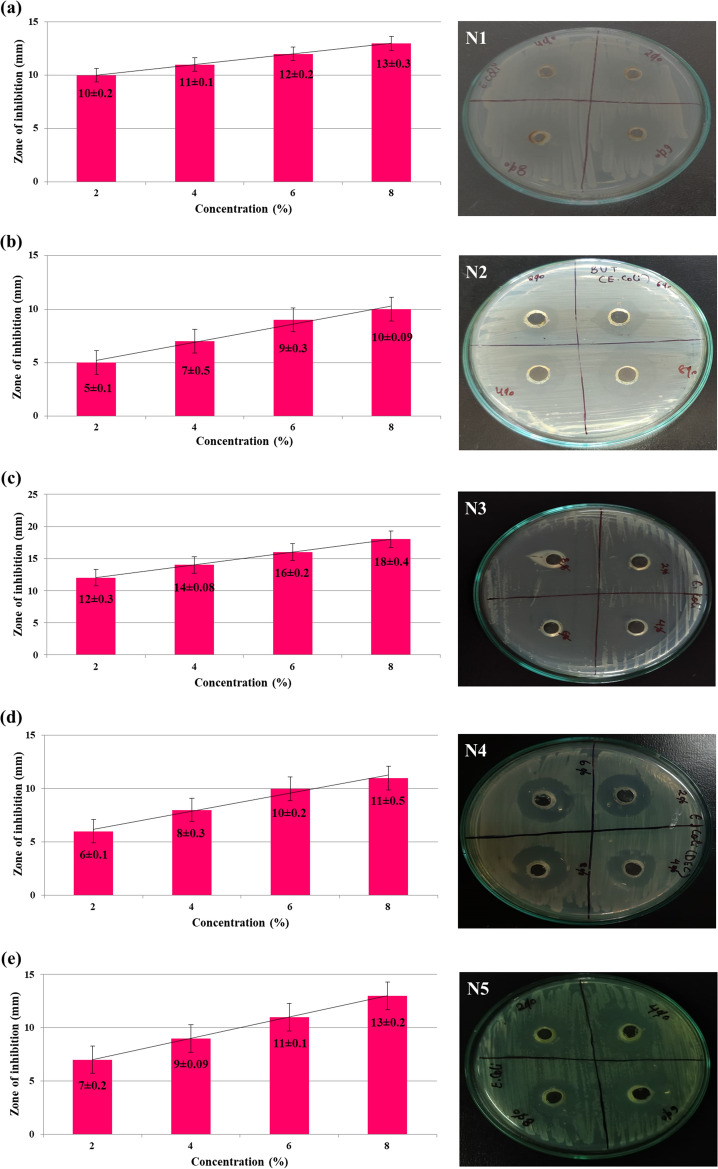
Depiction of zone of inhibition of acrylonitrile adducts against *E. coli* obtained by agar-well method via culture plates and histograms showing relationship between sample concentration and zone of inhibition.

**Figure 5:**
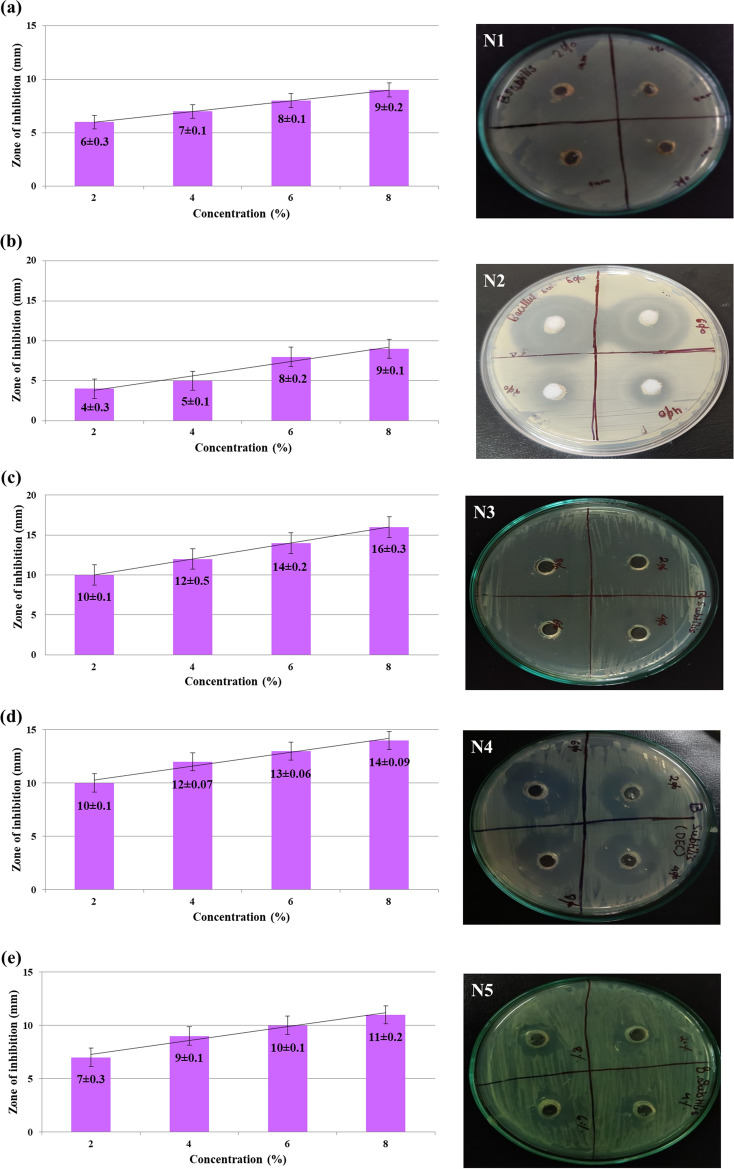
Zone of inhibition of acrylonitrile adducts against *B. subtilis* shown by agar-well method via culture plates and histograms showing relationship between sample concentration and zone of inhibition.

Zones of inhibition around each well and the results obtained for the inhibitory actions of acrylonitrile adducts against *E. coli* and *B. subtilis* are shown in Figs. 4 and 5 respectively which strongly shows its efficiency as a potential antibacterial agent. However, the negative control (distilled water) showed no antibacterial activity while the positive control (ceftriaxone) showed the zone of inhibition against *E. coli* (18 ± 0.1) and *B. subtilis* (17 ± 0.3 mm) (Fig. S4).

In case of *E. coli*, the diameter of the zone of inhibition for N1 at 2%, 4%, 6% and 8% are 10 ± 0.2 mm, 11 ± 0.1 mm, 12 ± 0.2 mm and 13 ± 0.3 mm respectively as shown in Fig. 4a. For N2, the diameter of the zone of inhibition at 2%, 4%, 6% and 8% are 5 ± 0.1 mm, 7 ± 0.5 mm, 9 ± 0.3 mm and 10 ± 0.09 mm as shown in Fig. 4b. From Fig. 4c, it was observed that the diameter of the zone of inhibition for N3 at 2%, 4%, 6% and 8% are 12 ± 0.3 mm, 14 ± 0.08 mm, 16 ± 0.2 mm, 18 ± 0.4 mm. For N4, the diameter of the zone of inhibition at 2%, 4%, 6% and 8% are 6 ± 0.1 mm, 8 ± 0.3 mm, 10 ± 0.2 mm and 11 ± 0.5 mm as shown in Fig. 4d. Similarly for N5, the diameter of the zone of inhibition at 2%, 4%, 6% and 8% are 7 ± 0.2 mm, 9 ± 0.09 mm, 11 ± 0.1 mm and 13 ± 0.2 mm respectively as shown in Fig. 4e.

Similarly, in case of *B. subtilis*, the diameter of the zone of inhibition for N1 at 2%, 4%, 6% and 8% are 6 ± 0.3 mm, 7 ± 0.1 mm, 8 ± 0.1 mm and 9 ± 0.2 mm respectively as shown in Fig. 5a. For N2, the diameter of the zone of inhibition at 2%, 4%, 6% and 8% are 4 ± 0.3 mm, 5 ± 0.1 mm, 8 ± 0.2 mm and 9 ± 0.1 mm as shown in Fig. 5b. From Fig. 5c, it was observed that the diameter of the zone of inhibition for N3 at 2%, 4%, 6% and 8% are 10 ± 0.1 mm, 12 ± 0.5 mm, 14 ± 0.2 mm, 16 ± 0.3 mm. For N4, the diameter of the zone of inhibition at 2%, 4%, 6% and 8% are 10 ± 0.1 mm, 12 ± 0.07 mm, 13 ± 0.06 mm and 14 ± 0.09 mm as shown in Fig. 5d. Similarly for N5, the diameter of the zone of inhibition at 2%, 4%, 6% and 8% are 7 ± 0.3 mm, 9 ± 0.1 mm, 10 ± 0.1 mm and 11 ± 0.2 mm respectively as shown in Fig. 5e.

The result of this study strongly reveals the antibacterial activity of acrylonitrile adducts at low, medium and high dose as shown in Tables 1 and 2. The diameter of the zone of inhibition here clearly demonstrates the effectiveness of the five acrylonitrile adducts as a potential antibacterial agent. The minimum inhibitory concentration was also evaluated for the five adducts as shown in Table 3.

**Table 1:** Zone of inhibition (mean standard error) for *E. coli*.

Acrylonitrile adducts	Low dose	Low dose	Medium dose	High dose
	2 mg	4 mg	6 mg	8 mg
N1	10 ± 0.2	11 ± 0.1	12 ± 0.2	13 ± 0.3
N2	5 ± 0.1	7 ± 0.5	9 ± 0.3	10 ± 0.09
N3	12 ± 0.3	14 ± 0.08	16 ± 0.2	18 ± 0.4
N4	6 ± 0.1	8 ± 0.3	10 ± 0.2	11 ± 0.5
N5	7 ± 0.2	9 ± 0.09	11 ± 0.1	13 ± 0.2

**Table 2:** Zone of inhibition (mean standard error) for *B. subtilis*.

Acrylonitrile adducts	Low dose	Low dose	Medium dose	High dose
	2 mg	4 mg	6 mg	8 mg
N1	6 ± 0.3	7 ± 0.1	8 ± 0.1	9 ± 0.2
N2	4 ± 0.3	5 ± 0.1	8 ± 0.2	9 ± 0.1
N3	10 ± 0.1	12 ± 0.5	14 ± 0.2	16 ± 0.3
N4	10 ± 0.1	12 ± 0.07	13 ± 0.06	14 ± 0.09
N5	7 ± 0.3	9 ± 0.1	10 ± 0.1	11 ± 0.2

**Table 3:** Minimal inhibitory concentration (MIC, μl/ml) of acrylonitrile adducts against Gram negative and Gram positive bacteria.

Organism	MIC (μl/ml)				
	N1	N2	N3	N4	N5
*Escherichia coli* (G−)	1	3	0.4	1	3
*Bacillus subtilis* (G+)	6	6	0.9	0.5	5

Similar to this study, novel nitriles and imidazolines were prepared and their antimicrobial activity was checked against Gram-positive (*Bacillus megaterium* and *Staphylococcus aureus*) and Gram-negative bacteria (*Escherichia coli* and *Proteus vulgaris*)^55^. In addition, the antifungal activity (*Aspergillus niger*) was also checked. They reported that for the gram-positive bacteria the diameter of the zone of inhibition was observed to be in the range of 10 to 21 mm while for the gram-negative bacteria the same was obtained in the range of 10 to 22 mm. In case of *A. niger*^55^ the zone of inhibition was observed to be in the range of 10 to 18 mm. In another study, Ammar et al*.*^56^ checked the antimicrobial activity of sulfonamide containing pyrazolopyrimidine, pyrimidine and pyrazolotriazine derivatives. Only two compounds showed good antibacterial activity (zone over 3.0 mm) against *Serratia marescens* and *Proteus mirabilis* while the rest of the compounds were showing antibacterial activity in the range of 0.6 to 1.4 mm^56^.

Dongre and Hadda^57^ reported better antibacterial activity of synthetic pyrido [2,3-d] pyrimidines armed with nitrile group against *Staphylococcus*, *Bacillus cereus*, *P. merabitis* and *S. maresens*^57^. Similarly, Aires et al*.*^58^, investigated the antimicrobial activity of isothiocyanates and indole-3-acetonitrile against gram-negative bacteria. They have also found that isothiocynates SFN and BITC have significant antibacterial activity and might be a potential compound for control of human pathogens through the diet^58^.

**Figure 6:**
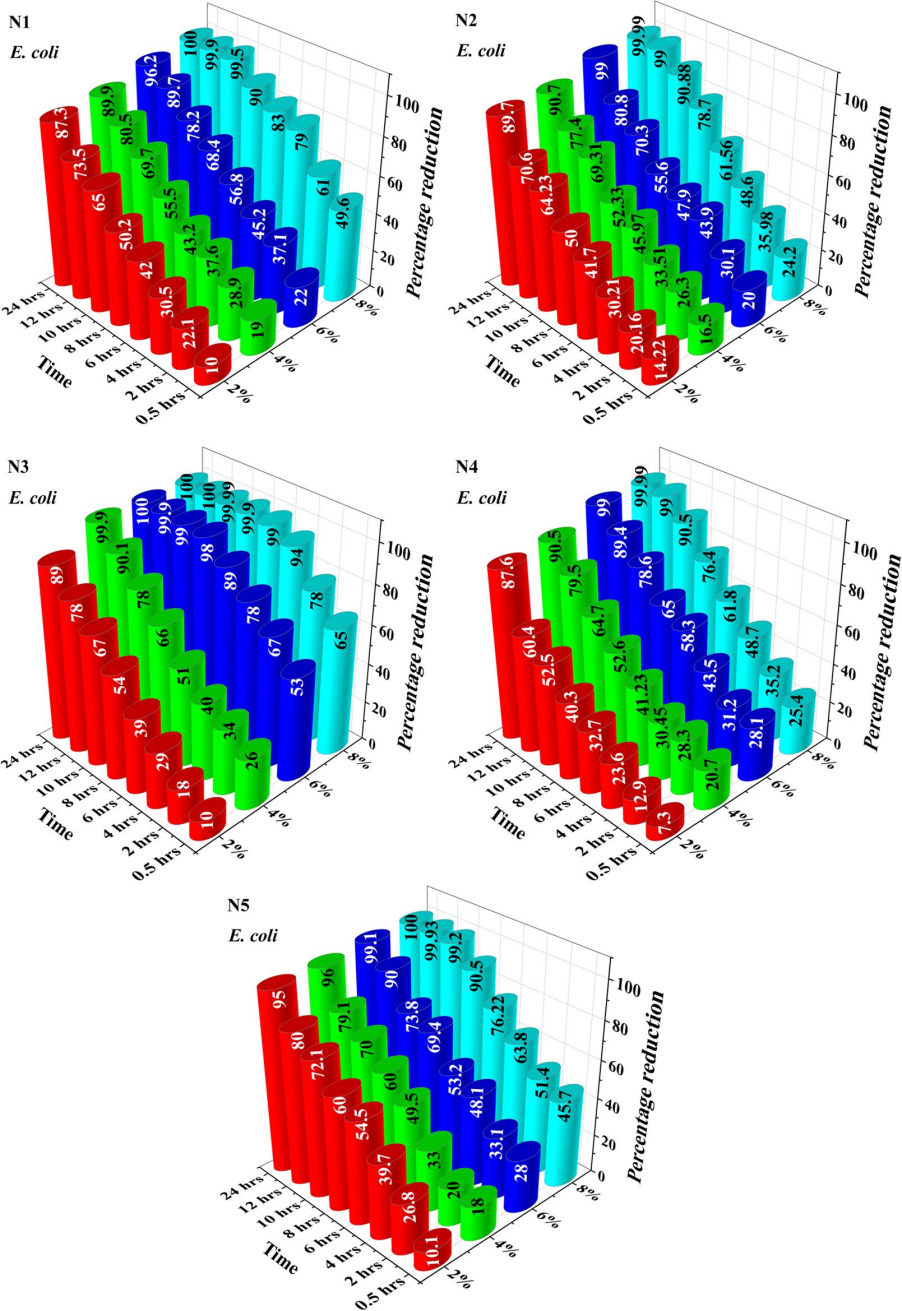
Percentage reduction of *E. coli* by acrylonitrile adducts.

**Figure 7:**
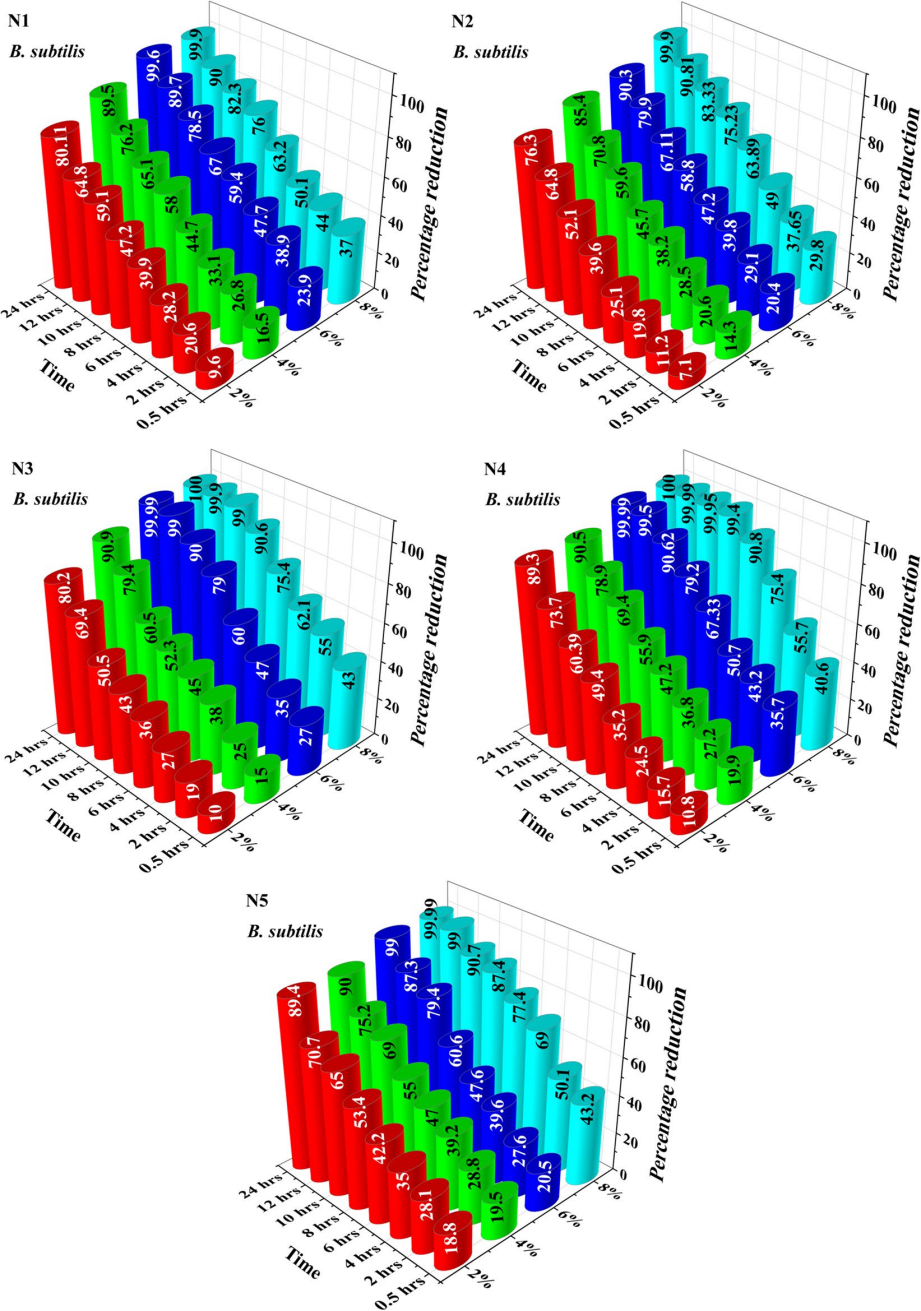
Percentage reduction of *B. subtilis* by acrylonitrile adducts.

The bactericidal activity of acrylonitrile adducts against *E. coli* and *B. subtilis* were also assessed by the time-kill kinetic analysis^59^. Any reduction in bactericidal activity was evaluated by determination of the bacterial survival rate when exposed to acrylonitrile adducts. The percentage reduction of *E. coli* and *B. subtilis* are shown in Figs. 6 and 7 respectively. The result of this study revealed that after 24 h, all the bacterial cells of *E. coli* and *B. subtilis* were removed by acrylonitrile adducts and thereby confirming its bactericidal nature^60^.

In case of *E. coli*, the 2% of n-propyliminobis-propionitrile (N1), n-butyliminobis-propionitrile (N2), n-hexyliminopropionitrile (N3), n-decyliminopropionitrile (N4) and n-dodecyliminopropionitrile (N5) removed the *E. coli* with reduction of 10, 14.22, 10, 7.3 and 10.1 percentage respectively at 0.5 h. On further increasing the incubation time the percentage reduction at 24 h against *E. coli* for N1, N2, N3, N4 and N5 was recorded as 87.3%, 89.7%, 89%, 87.6% and 95% respectively. On increasing the amount to 4%, antibacterial activity increased from 19 to 89.9%, 16.5 to 90.7%, 26 to 99.9%, 20.7 to 90.5% and 18 to 96% for N1, N2, N3, N4 and N5, from 0.5 to 24 h respectively. On further increasing the amount to 6%, the percentage reduction was observed as 22 to 96.2%, 20 to 99%, 53 to 99.999%, 28.1 to 99%, 28 to 99.1% for N1, N2, N3, N4 and N5 from 0.5 to 24 h respectively. When the concentration of acrylonitrile adducts was further increased to 8%, the percentage reduction was found to be 100, 99.99, 100, 99.99 and 100% against *E. coli* after incubation time of 24 h with 100 ml of media.

In case of *B. subtilis*, the 2% of N1, N2, N3, N4 and N5 removed *B. subtilis* with reduction of 9.6, 7.1, 10, 10.8 and 18.8 percentage respectively at 0.5 h. Increasing the incubation time further to 24 h, the percentage reduction against *B. subtilis* was increased to 80.11%. On increasing the amount of N1, N2, N3, N4 and N5 to 4%, antibacterial activity increases from 16.5 to 89.5%, 14.3 to 85.4%, 15 to 90.9%, 19.9 to 90.5% and 19.5 to 90%, from 0.5 to 24 h respectively. On further increasing the amount to 6%, the percentage reduction was found to increase from 23.9 to 99.6%, 20.4 to 90.3%, 27 to 99.99%, 35.7 to 99.99% and 20.5 to 99% for N1, N2, N3, N4 and N5, from 0.5 to 24 h respectively. Finally, further increase in concentration of N1, N2, N3, N4 and N5 to 8%, significant change of percentage reduction was noted which were recorded as 99.9, 99.9, 100, 100 and 99.99 percentage reduction against *B. subtilis*, after incubation for 24 h with 100 ml of media.

The results of antibacterial activity of both bacteria under dynamic condition reveals that all the bacterial cells were removed by N1, N2, N3, N4 and N5 after contacting for 24 h.

**Figure 8:**
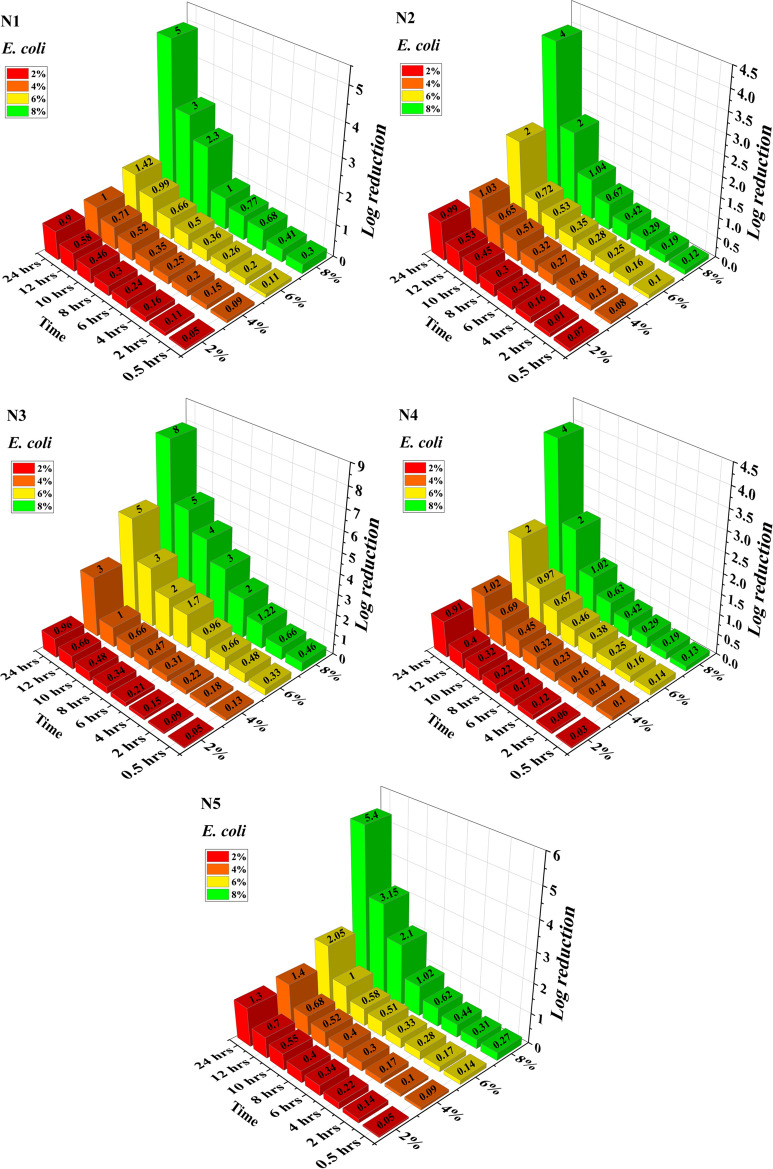
Log reduction of *E. coli* by acrylonitrile adducts.

**Figure 9:**
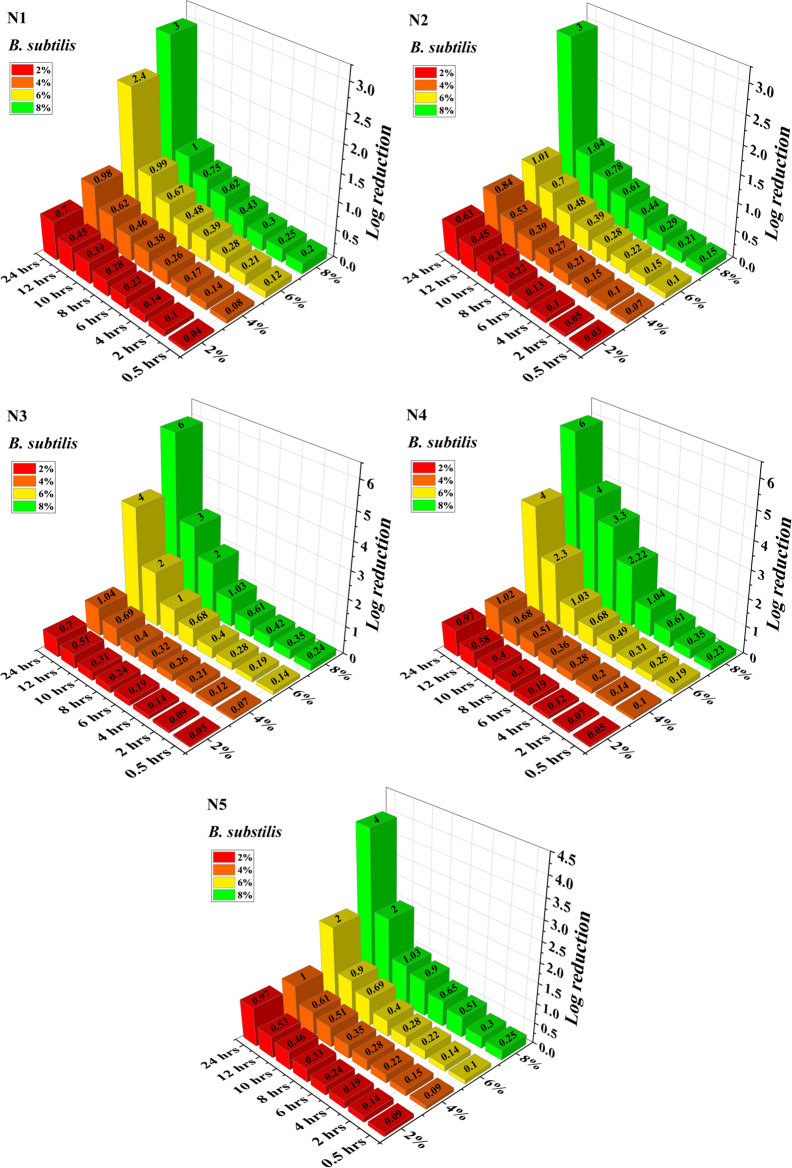
Log reduction of *B. subtilis* by acrylonitrile adducts.

Figures 8 and 9 shows the Log reduction (log_10_ cfu/ml) values for *E. coli* and *B. subtilis*. It is mathematical term used to express the relative count of living microbes eliminated by antibiotic. For instance, if the number of cfus in the control sample was found to be 10^6^ and after using antibiotic the test sample was having 10^5^, that would be a Log reduction of 1 or a reduction of 90%. In the present study, the bactericidal activity of acrylonitrile adducts were evaluated by the time-kill kinetic analysis. The reduction in bactericidal activity was evaluated by determination of the survival rate [log_10_ cfu/ml vs Time(h)] of bacteria when exposed to a drug.

At 2% v/v N1, N2, N3, N4 and N5 treatment, the cell population was reduced by 0.05, 0.07, 0.05, 0.03 and 0.05 for *E. coli* respectively and for *B. subtilis* it was reduced by 0.04, 0.03, 0.05, 0.05 and 0.09, at 0.5 h. While at 4% v/v N1, N2, N3, N4 and N5 treatment, the considerable growth inhibitory response was observed at 24 h in both Gram positive (1 log10 cfu/ml) and Gram negative (3 log10cfu/ml) bacteria. On further increasing the concentration of N1, N2, N3, N4 and N5 to 6% v/v, log reduction observed for *E. coli* was 1.42, 2, 5, 2 and 2.05 log cfu/ml respectively and the corresponding values for *B. subtilis* was noted as 2.4, 1.01, 4, 4 and 2 log cfu/ml respectively. Increasing the concentration further to 8% (V/V), log reduction values were dramatically improved to 5, 4, 8, 4 and 5.4 log cfu/ml in case of *E. coli* while in case of *B. subtilis* the values were obtained as 3, 3, 6, 6 and 4 log cfu/ml reduction. Based on the growth pattern, the action of 2%, 4%, 6% and 8% v/v on N1, N2, N3, N4 and N5 indicates a bactericidal effect.

### Biological studies

**Figure 10:**
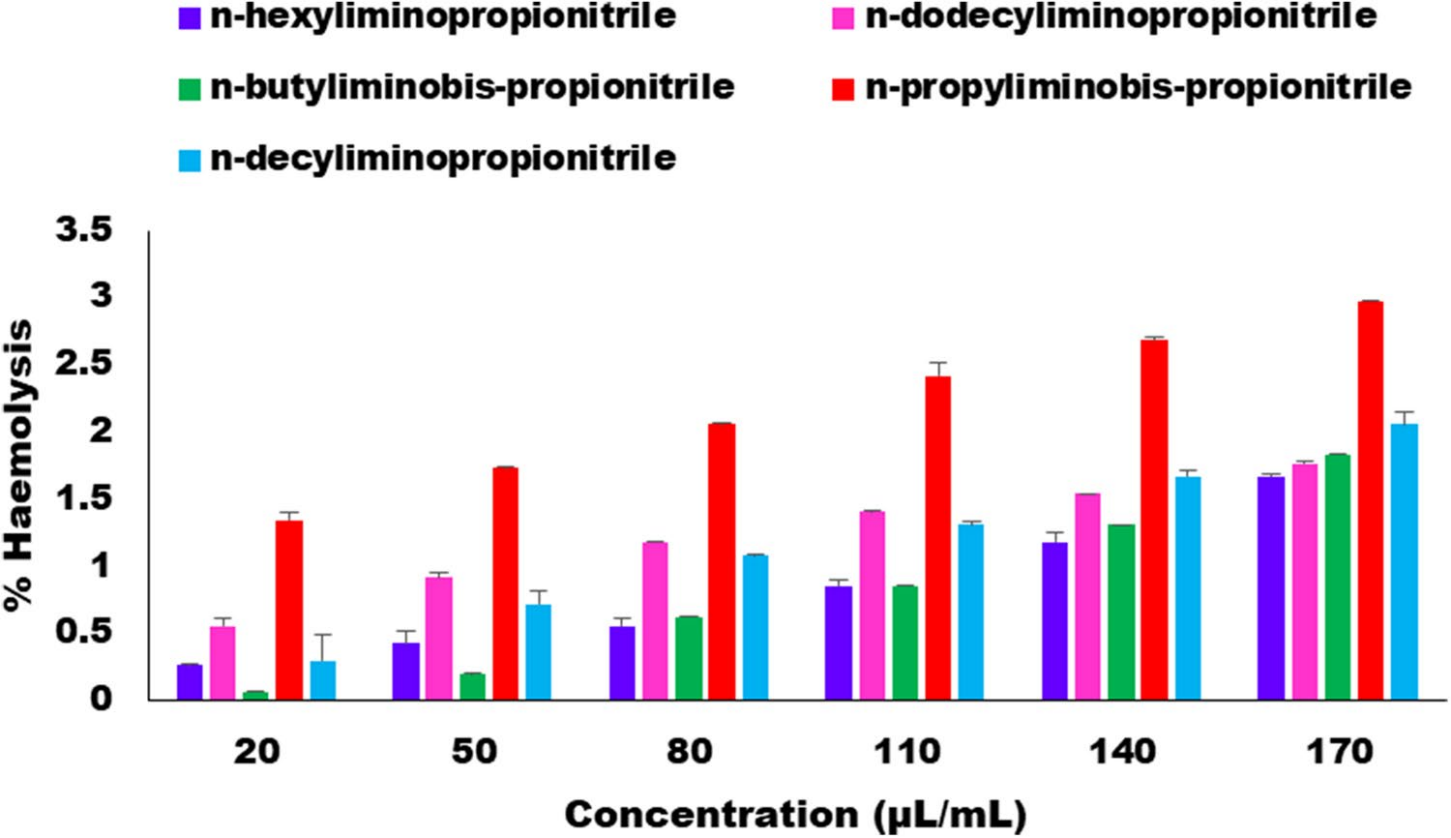
%Haemolysis of acrylonitrile adducts at different concentrations.

In the present study five acrylonitrile adducts such as n-propyliminobis-propionitrile (N1), n-butyliminobis-propionitrile (N2), n-hexyliminopropionitrile (N3), n-decyliminopropionitrile (N4) and n-dodecyliminopropionitrile (N5) were used to check its haemolytic activity with normal erythrocytes of mouse. Haemolysis is a process occurring as a result of contact of blood with a foreign object and as per the standards of American Society for Testing and Materials (ASTM) if the percentage of haemolysis is < 5% then it is considered as no haemolysis^61^, 5–10% low haemolysis and > 10% marked haemolysis^62^. In vitro biocompatibility assessment of acrylonitrile adducts were carried out by direct contact with erythrocytes using PBS and the %haemolysis is shown in Fig. 10.

The positive control and negative control in this study was 1% TritonX-100 and 1× PBS respectively and their absorbance is mentioned in Table 4. According to this table the %haemolysis of all the synthesized compounds ranged between 0.06 and 2.97% relative to normal mouse erythrocytes at various concentration exhibiting no adverse effects of the compounds. However, out of 5 acrylonitrile adducts synthesised the n-hexyliminopropionitrile showed the least % haemolysis (0.26% to 1.66%) at various concentration (20 to 170 µl/ml) and thus safe for clinical trials.

**Table 4:** Percentages of haemolytic activity of five acrylonitrile adducts.

Comp	N1		N2		N3		N4		N5	
Conc. (µl/ml)	Abs. (A_t_)	%H	Abs. (A_t_)	%H	Abs. (A_t_)	%H	Abs. (A_t_)	%H	Abs. (A_t_)	%H
20	0.0197	1.33 ± 0.06	0.0158	0.06 ± 0.004	0.0164	0.26 ± 0.01	0.0165	0.29 ± 0.2	0.0173	0.55 ± 0.06
50	0.0209	1.73 ± 0.004	0.0162	0.19 ± 0.006	0.0169	0.42 ± 0.09	0.0178	0.71 ± 0.09	0.0184	0.91 ± 0.04
80	0.0219	2.05 ± 0.007	0.0175	0.62 ± 0.002	0.0173	0.55 ± 0.06	0.0189	1.07 ± 0.01	0.0192	1.17 ± 0.01
110	0.023	2.41 ± 0.09	0.0182	0.84 ± 0.007	0.0182	0.84 ± 0.05	0.0196	1.30 ± 0.03	0.0199	1.40 ± 0.009
140	0.0238	2.67 ± 0.03	0.0196	1.30 ± 0.003	0.0192	1.17 ± 0.08	0.0207	1.66 ± 0.04	0.0203	1.53 ± 0.005
170	0.0247	2.97 ± 0.008	0.0212	1.83 ± 0.003	0.0207	1.66 ± 0.02	0.0219	2.05 ± 0.09	0.021	1.76 ± 0.02

% H = % Haemolysis; Abs. (A_t_) = Absorbance of test sample; Positive control (A_s_) = 0.306, Negative control (A_n_) = 0.0156.

Shanmugam et al.^63^ synthesized pentasubstituted pyrroles and subjected to in vitro anti-thrombotic analysis. The divergent pyrroles 2-(2-argio-1-methyl-5-(methylthio)-4-nitro1H-pyrrol-3-yl)acetonitrile derivatives exhibited excellent clot lysis. Danac et al*.*^64^ has synthesized cyano-substituted pyrrole fused (iso)quinolone derivatives. Anticancer study showed that fumaronitrile has potent anticancer properties. It exhibited antiproliferative action against cancer cell lines. The thrombolytic activity was evaluated and it was found that the blood dissolving activity was less than the negative control so the adducts did not showed any thrombolytic activity as shown in Table 5.

**Table 5:** Percentages of thrombolytic activity of five acrylonitrile adducts.

Compound/sample	Z = weight after clot lysis	$\text{\%}Thrombolysis=\frac{z}{y}\times 100$
Negative control*	1.21	31.3
Positive control**	1.82	90.6
N1	1.27	10.58
N2	1.37	4.9
N3	1.16	13.75
N4	1.19	10.6
N5	1.22	10.6

Weight of clot before lysis (y) = 2.15 g, dis. *DMSO, **Streptokinase (SK).

## Conclusion

Successful synthesis of five acrylonitrile adducts have been demonstrated in this work, using environmentally benign synthetic method. The novelty of the compounds was exhibited in terms of their excellent antimicrobial activity against both the bacterial stains. Most importantly, during the study, the reported compounds were also established as potent haemolytic agent, which significantly enhances the pharmaceutical and biological significance of the compounds. All these compounds showed least percentage of haemolysis, 0.06 to 2.97%, which is safe for clinical trials. This fact other way indicates that the acrylonitrile adducts successfully can be employed for drug delivery. Study on the bactericidal characteristics of the compounds indicated that for all the five acrylonitrile adducts, complete removal of both the gram-positive and gram-negative bacteria were possible with an 8% concentration of the compounds, after 24 h, which strongly reveals the bactericidal characteristics of the compounds. Summing up, we have developed a series of acrylonitrile adducts which can be explored as efficient antimicrobial and haemolytic agent.

## Supplementary Information

Supplementary Information.

## Data Availability

The datasets used and/or analysed during the current study are available from the corresponding author on reasonable request.
