# Supplementary material for: Acrylonitrile adducts: design, synthesis and biological evaluation as antimicrobial, haemolytic and thrombolytic agent
Source: Sci Rep. 2023 Apr 17;13:6209. doi: 10.1038/s41598-023-33605-1 (PMC10110592; doi:10.1038/s41598-023-33605-1)
Supplement: Supplementary file 1 — Supplementary Information. [file 41598_2023_33605_MOESM1_ESM.docx]

**Acrylonitrile adducts: Design, synthesis and biological evaluation as antimicrobial, haemolytic and thrombolytic agent**

Parineeta Das^[1]^, Nirmala Devi^[2]^, Nisha Gaur^[3]^, Swagata Goswami^[3]^, Dhiraj Dutta^[3]^, Rama Dubey^[3]^ and Amrit Puzari*^[1]^

^[1]^Department of Chemistry, National Institute of Technology Nagaland, Chumoukedima, Nagaland, India-797103

^[2]^Chulalongkorn University, Bangkok, Thailand-10330

^[3]^Defence Research Laboratory, Post Bag No. 2, Tezpur, Assam, India-784001

*Corresponding author. Dr. Amrit Puzari

Tel.: +91-03862-241813

E-mail: [amrit09us@yahoo.com](mailto:amrit09us@yahoo.com)

**Content Page No.**

General information 3

Spectral data acrylonitrile adducts 4-7

^1^H NMR spectra of acrylonitrile adducts 7-8

ESI-Mass spectra of acrylonitrile adducts 9-11

Antimicrobial activity 11-12

References 12-13

**General information**

All the chemicals used for the synthesis of mono and di-adducts of acrylonitrile were procured from Sigma-Aldrich and TCI Chemicals, Japan. Molecular sieves of 4Aº beads, 4-8 mesh were purchased from Sigma-Aldrich. Merck silica gel 60 F_254_ plates were used for thin layer chromatography. All the chemical reagents were of analytical grade and were used without further purification. Solvents used during the synthesis were dried by using standard procedure. Mixing of the substrates was carried out by LED Digital Vortex Mixer.

**General method for synthesis of** **acrylonitrile adducts**

n-alkyliminobis-propionitrile and n-alkyliminopropionitrile was synthesized by aza-Michael addition reaction under microwave irradiation using literature procedure[1] as shown in Figure S1. In this reaction, 2.5 mmol acrylonitrile was mixed with 1 mmol n-alkylamine in a test tube and 0.12 g of finely powdered molecular sieves (4Aº), which act as a catalyst, was added to the same. After proper mixing, it was subjected to microwave irradiation at 40 ºC for a period of 2 hours. After the reaction is completed, the content in the test tubes were cooled down to room temperature and the catalyst was removed by centrifugation at a speed of 2500 rpm for 5 minutes. The supernatant obtained was collected and concentrated under vacuum evaporation.

**Figure S1**. Synthetic route for acrylonitrile adducts

**Spectral data for the aza-Michael addition products**

**n-propyliminobis-propionitrile (N1, C_9_H_15_N_3_)** [1]

82.09 µL (1 mmol) of n-propylamine was dissolved in 164.57 µL (2.5 mmol) of acrylonitrile and to this 0.12 g molecular sieve was added. The reaction mixture was heated at 40 ^o^C for 2 h under microwave irradiation. The reaction was monitored with the help of thin layer chromatographic technique (30% ethyl acetate as eluent). On completion of reaction, it was cooled down to normal room temperature and catalyst was separated by centrifuge technique at 2500 r.p.m. for 5 minutes. The supernatant was collected and further purified by column chromatography. Upon isolation of product the solvents were evaporated. Recrystallization from methanol afforded 163.19 g (98.9 %) **N1**. B.p.: 310-313 ^0^C; R_f_ = 0.7; ^1^H NMR (300 MHz, CDCl_3_): *δ* = 0.94 (t, 3H, *J* = 7.5 Hz, CH_3_ terminal), 1.42-1.54 {m, 2H, *J* = 7.5 Hz, CH_2_ (propyl)}, 2.47 {t, 2H, *J* = 2.4 Hz, CH_2_−N (propyl)}, 2.52 (t, 2H, *J* = 7.2 Hz, CH_2_−CN), 2.83 (t, 2H, *J* = 6.9 Hz, CH_2_−N) ppm; ^13^C{^1^H} NMR (300 MHz, CDCl_3_): *δ* = 11.6(−CH_3_), 20.7(−CH_2_−), 118 (C≡N) ppm; IR: ν_max_ = 2245 (C≡N stretching), 2825-2961 (C−H stretching), 1136 (C−N stretch) cm^-1^; MS: *m*/z = 165 (M^+^), calcd for C_9_H_15_N_3_ 165, found = 166.4.

**n-butyliminobis-propionitrile (N2, C_10_H_17_N_3_)** [1]

99.1 µL (1 mmol) of n-butylamine was dissolved in 164.57 µL (2.5 mmol) of acrylonitrile and to this 0.12 g molecular sieve was added. The reaction mixture was heated at 40 ^o^C for 2 h under microwave irradiation. The reaction was monitored with the help of thin layer chromatographic technique (30% ethyl acetate as eluent). On completion of reaction, it was cooled down to normal room temperature and catalyst was separated by centrifuge technique at 2500 r.p.m. for 5 minutes. The supernatant was collected and further purified by column chromatography. Upon isolation of product the solvents were evaporated. Recrystallization from methanol afforded 176.673g (98.7%) **N2**. B.p.: 366-368 ^0^C; R_f_ = 0.79; ^1^H NMR (300 MHz, CDCl_3_): *δ* = 0.91 (t, 3H, *J* = 3 Hz, CH_3_ terminal), 1.33 {m, 2H, *J* = 3.3 Hz, CH_2_ (butyl)}, 1.39 {m, 2H, *J* = 3.9 Hz, CH_2_ (butyl)}, 2.42 {t, 2H, *J* = 6.6 Hz, CH_2_−NH (butyl)}, 2.5 (t, 2H, *J* = 6.9 Hz, CH_2_−CN), 2.8 {t, 2H, *J* = 6.6 Hz CH_2_−NH (propyl)} ppm; ^13^C{^1^H} NMR (300 MHz, CDCl_3_): *δ* = 13.8 (−CH_3_), 30.5 (−CH_2_−), 118 (C≡N) ppm; IR: ν_max_ = 2247 (C≡N stretching), 2860-2957 (C−H stretching), 1127 (C−N stretch) cm^-1^; MS: *m*/z = 179 (M^+^), calcd for C_10_H_17_N_3_ 179, found = 180.

**n-hexyliminopropionitrile (N3, C_9_H_18_N_2_)** [1]

132.27 µL (1 mmol) of n-hexylamine was dissolved in 164.57 µL (2.5 mmol) of acrylonitrile and to this 0.12 g molecular sieve were added. The reaction mixture was heated at 40 ^o^C for 2 h under microwave irradiation. The reaction was monitored with the help of thin layer chromatographic technique (30% ethyl acetate as eluent). On completion of reaction, it was cooled down to normal room temperature and catalyst was separated by centrifuge technique at 2500 r.p.m. for 5 minutes. The supernatant was collected and further purified by column chromatography. Upon isolation of product the solvents were evaporated. Recrystallization from methanol afforded 151.382 g (98.3%) **N3**. B.p.: 250-252 ^0^C; R_f_ = 0.77; ^1^H NMR (300 MHz, CDCl_3_): *δ* = 0.88 (t, 3H, *J* = 6.9 Hz CH_3_ terminal), 1.29 {m, 2H, CH_2_ (hexyl)}, 2 (m, 1H, NH), 2.53 {t, 2H, *J* = 6.6 Hz, CH_2_−NH (butyl)}, 2.8 {t, 2H, *J* = 6.6 Hz, CH_2_−NH (propyl)}, 2.9 (t, 2H, *J* = 6.6 Hz, CH_2_−CN) ppm. ^13^C{^1^H} NMR (300 MHz, CDCl_3_): *δ* = 13.8 (−CH_3_), 31.2 (−CH_2_−), 118 (C≡N) ppm; IR: ν_max_ = 2251 (C≡N stretching), 2853-2925 (C−H stretching), 1127 (C−N stretch) cm^-1^; MS: *m*/z = 154 (M^+^), calcd for C_9_H_18_N_2_ 154, found = 155.3.

**n-decyliminopropionitrile (N4, C_13_H_26_N_2_)** [1]

199.87 µL (1 mmol) of n-decylamine was dissolved in 164.57 µL (2.5 mmol) of acrylonitrile and to this 0.12 g molecular sieve was added. The reaction mixture was heated at 40 ^o^C for 2 h under microwave irradiation. The reaction was monitored with the help of thin layer chromatographic technique (30% ethyl acetate as eluent). On completion of reaction, it was cooled down to normal room temperature and catalyst was separated by centrifuge technique at 2500 r.p.m. for 5 minutes. The supernatant was collected and further purified by column chromatography. Upon isolation of product the solvents were evaporated. Recrystallization from methanol afforded 205.8 g (98%) **N4**. B.p.: 520-523 ^0^C; R_f_ = 0.73; ^1^H NMR (300 MHz, CDCl_3_): *δ* = 0.88 (t, 3H, *J* = 6.9 Hz, CH_3_ terminal), 1.26 {m, 2H, CH_2_ (decyl)}, 2 (m, 1H, NH), 2.53 {t, 2H, *J* = 6.6 Hz CH_2_−NH (decyl)}, 2.8 {t, 2H, *J* = 6.9 Hz, CH_2_−NH (propyl)}, 2.9 (t, 2H, *J* = 6.6 Hz, CH_2_−CN) ppm; ^13^C{^1^H} NMR (300 MHz, CDCl_3_): *δ* = 14 (−CH_3_), 31.8 (−CH_2_−), 118 (C≡N) ppm; IR: ν_max_ = 2247 (C≡N stretching), 2851-2922 (C−H stretching), 1129 (C−N stretch) cm^-1^; MS: *m*/z = 210 (M^+^), calcd for C_13_H_26_N_2_ 210, found = 211.

**n-dodecyliminopropionitrile (N5, C_15_H_30_N_2_)** [1]

229.97 µL (1 mmol) of n-dodecylamine was dissolved in 164.57 µL (2.5 mmol) of acrylonitrile and to this 0.12 g molecular sieve was added. The reaction mixture was heated at 40 ^o^C for 2 h under microwave irradiation. The reaction was monitored with the help of thin layer chromatographic technique (30% ethyl acetate as eluent). On completion of reaction, it was cooled down to normal room temperature and catalyst was separated by centrifuge technique at 2500 r.p.m. for 5 minutes. The supernatant was collected and further purified by column chromatography. Upon isolation of product the solvents were evaporated. Recrystallization from methanol afforded 233.24 g (98%) **N5**. B.p.: 530-532 ^0^C; R_f_ = 0.75; ^1^H NMR (300 MHz, CDCl_3_): *δ* = 0.88 (t, 3H, *J* = 6.9 Hz, CH_3_ terminal), 1.26 {m, 2H, CH_2_ (dodecyl)}, 2 (m, 1H, NH), 2.53 {t, 2H, *J* = 6.6 Hz, CH_2_−NH (dodecyl)}, 2.8 {t, 2H, *J* = 6.6 Hz, CH_2_−NH (propyl)}, 2.9 (t, 2H, *J* = 6.6 Hz, CH_2_−CN) ppm; ^13^C{^1^H} NMR (300 MHz, CDCl_3_): *δ* = 14.1 (−CH_3_), 31.9 (−CH_2_−), 118 (C≡N); IR: ν_max_ = 2251 (C≡N stretching), 2853-2920 (C−H stretching), 1129 (C−N stretch) cm^-1^; MS: *m*/z = 238 (M^+^), calcd for C_9_H_15_N_3_ 238, found = 239.

**^1^H NMR spectra of Michael adducts**


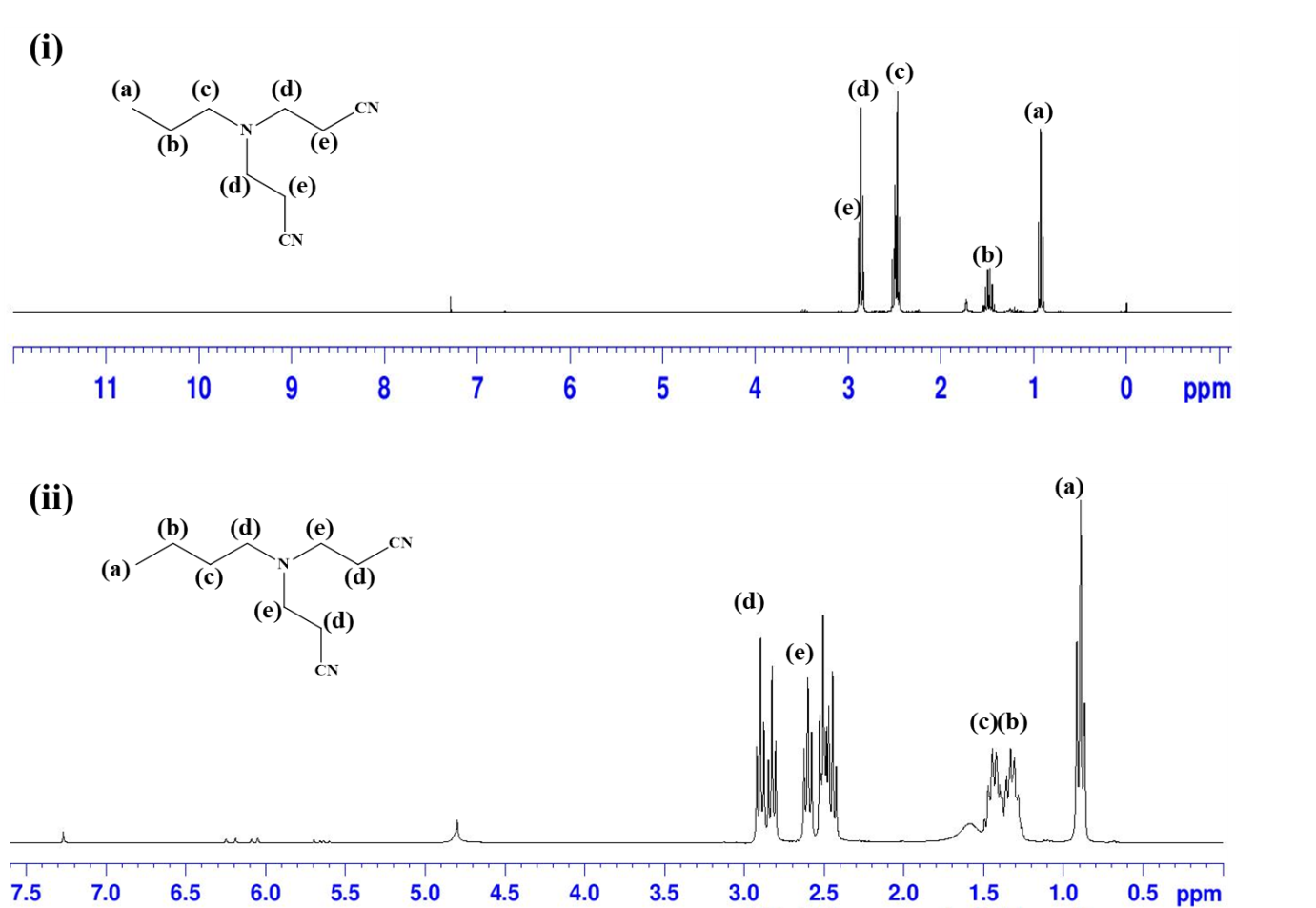


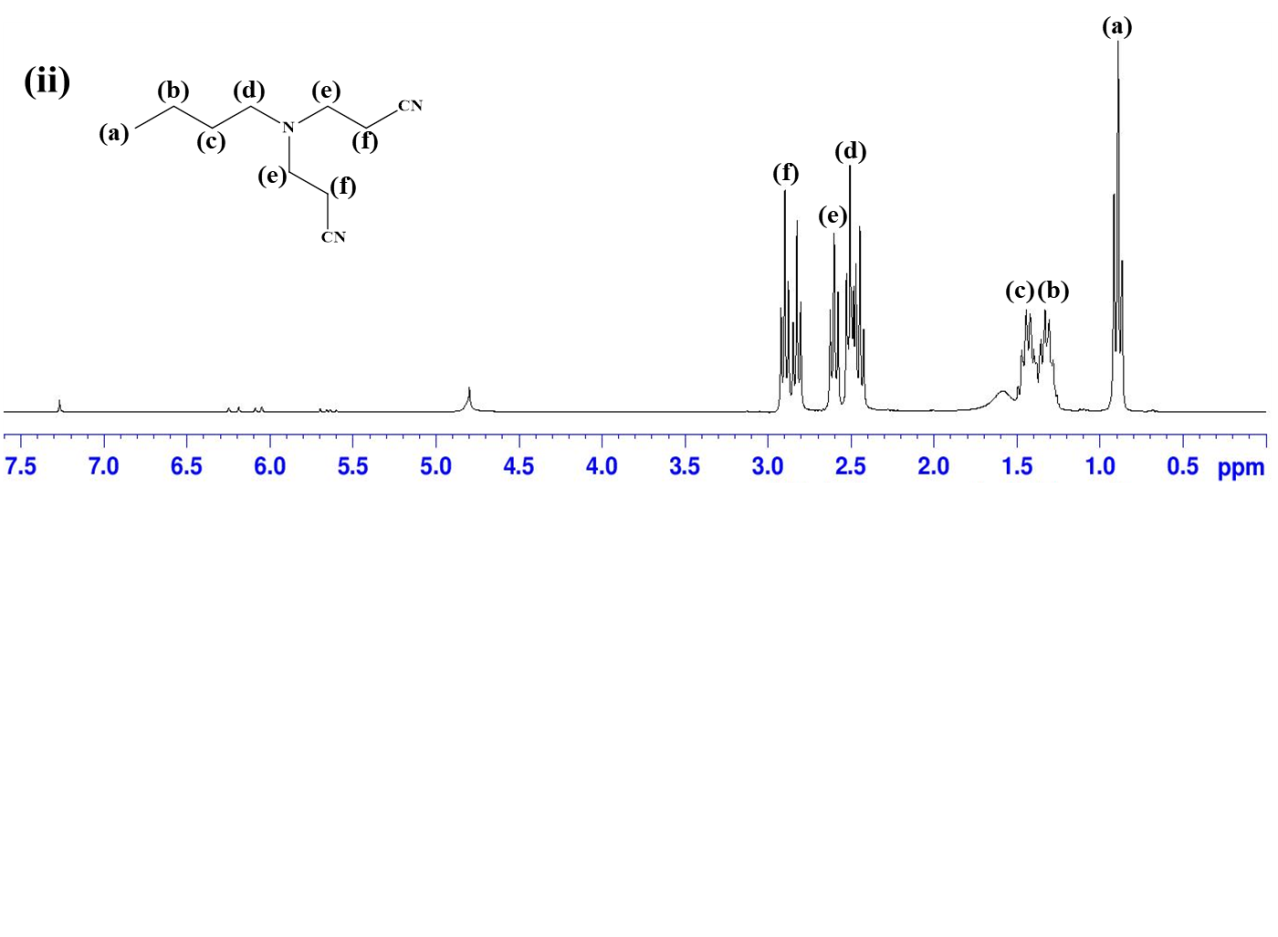


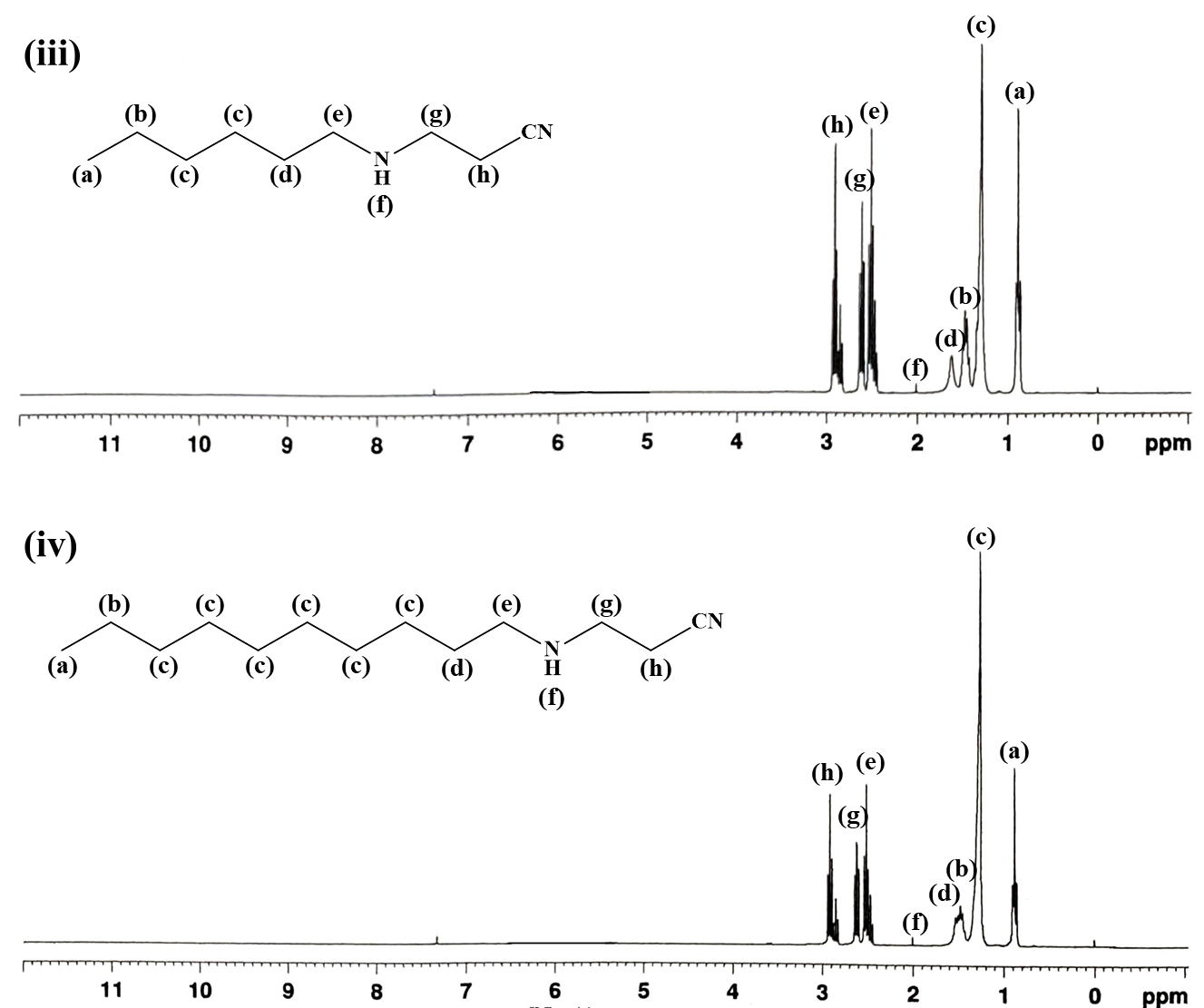


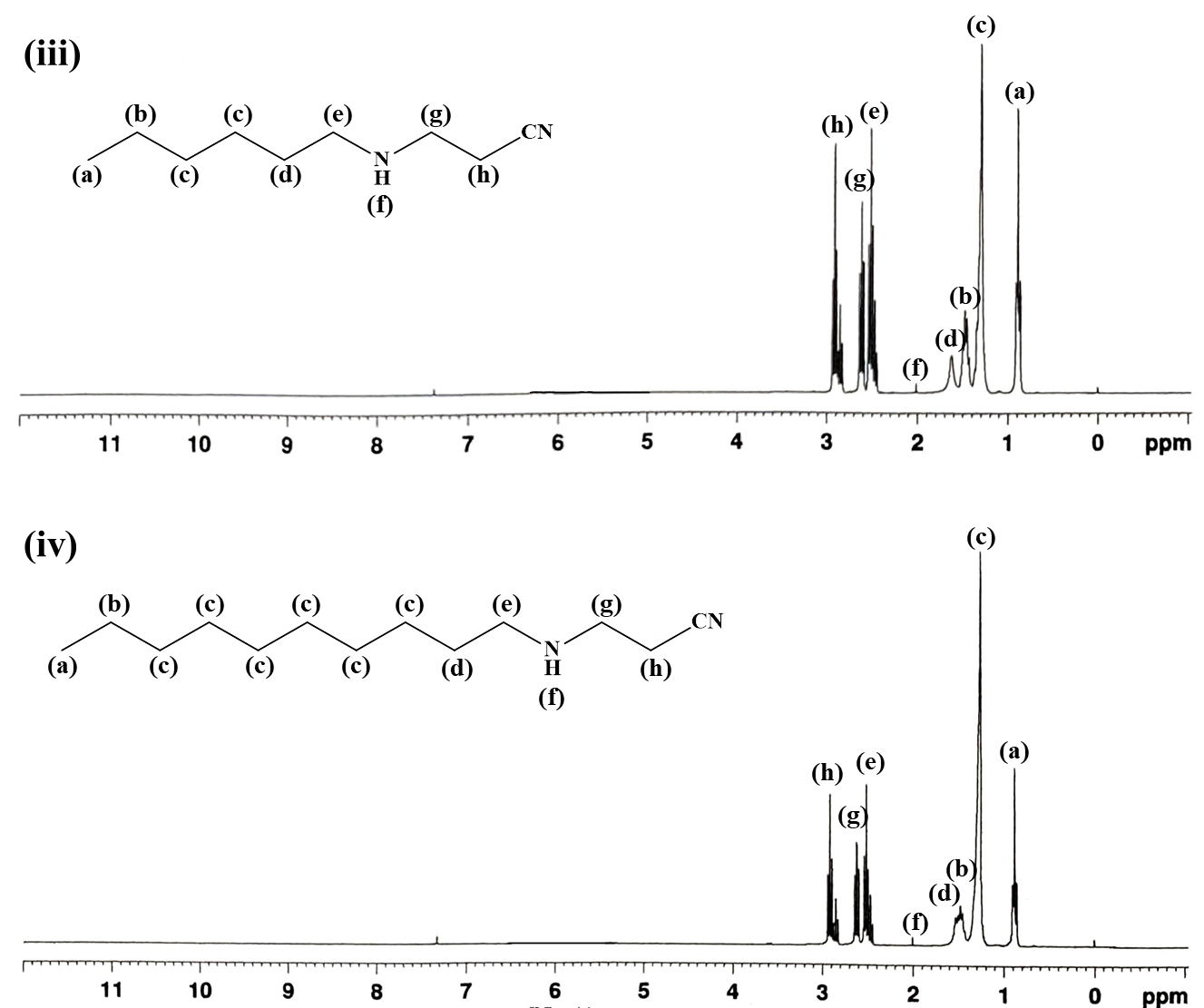


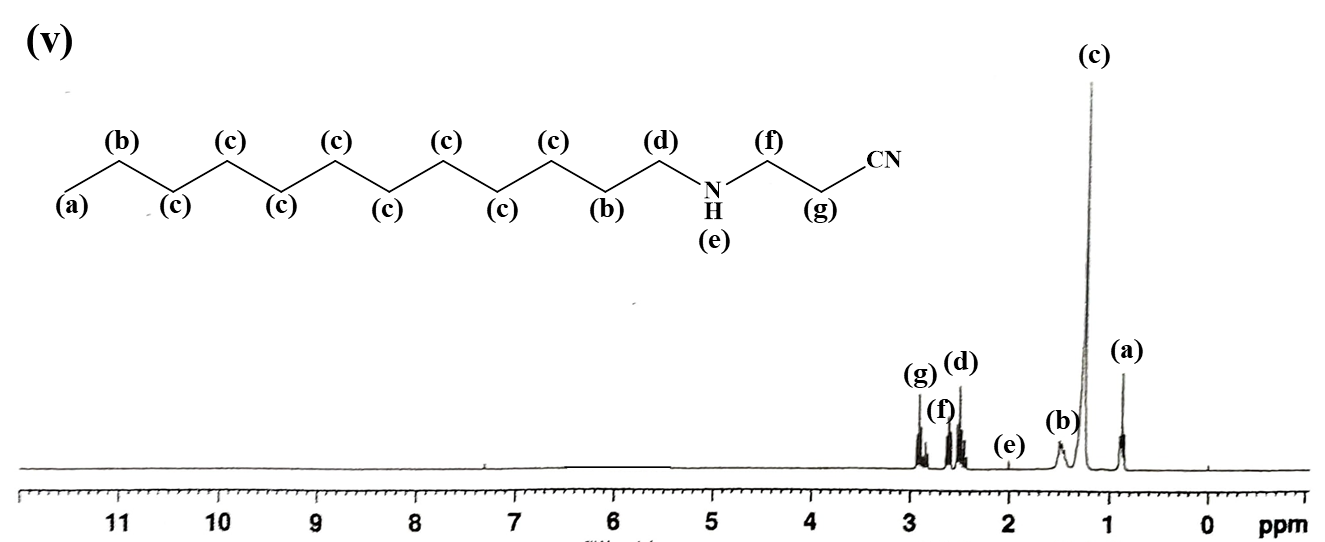


**Figure S2.** ^1^H NMR spectra of (i) n-propyliminobis-propionitrile (**N1**), (ii) n-butyliminobis-propionitrile (**N2**), (iii) n-hexyliminopropionitrile (**N3**), (iv) n-decyliminopropionitrile (**N4**) and (v) n-dodecyliminopropionitrile (**N5**) [1].

**ESI-Mass spectra of Michael adducts**

**
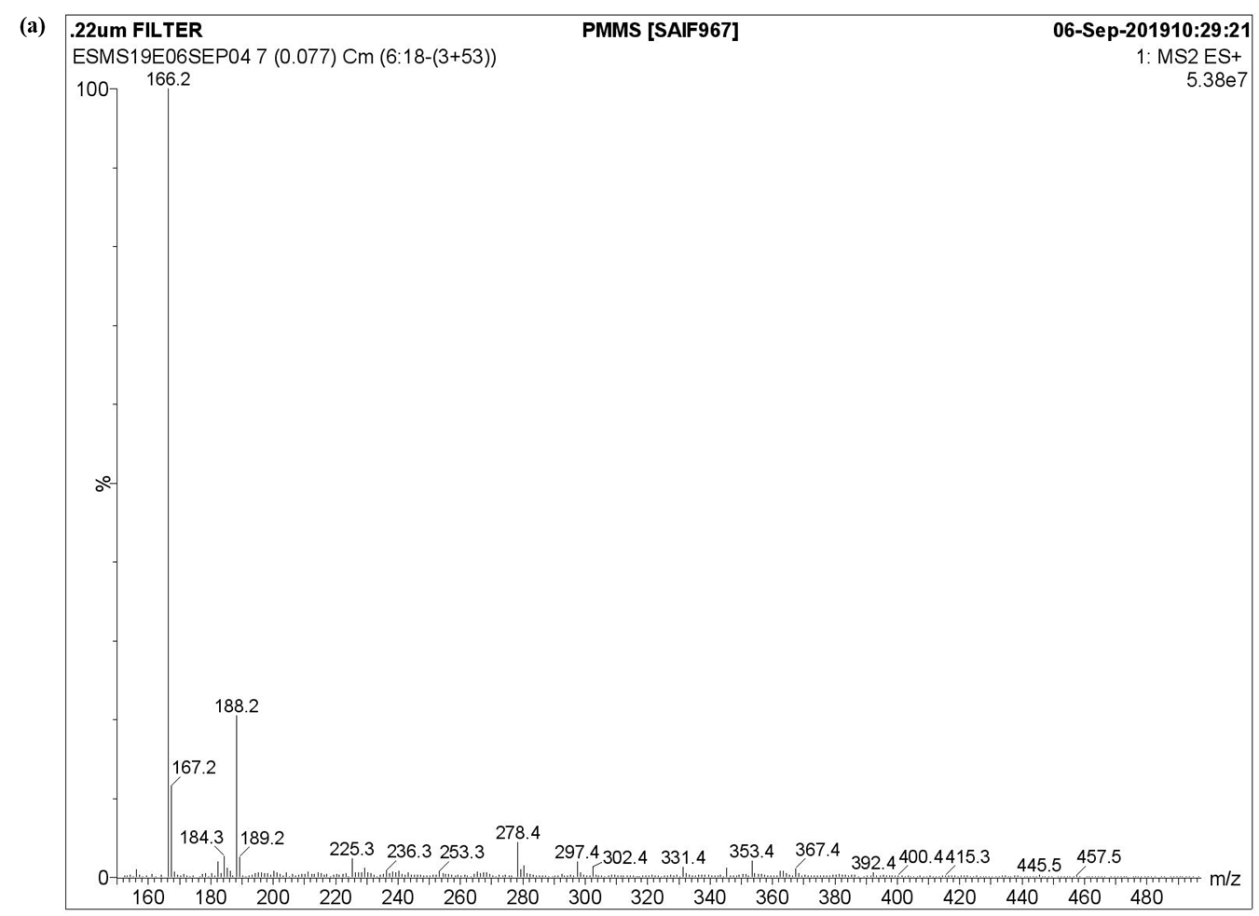
**

**
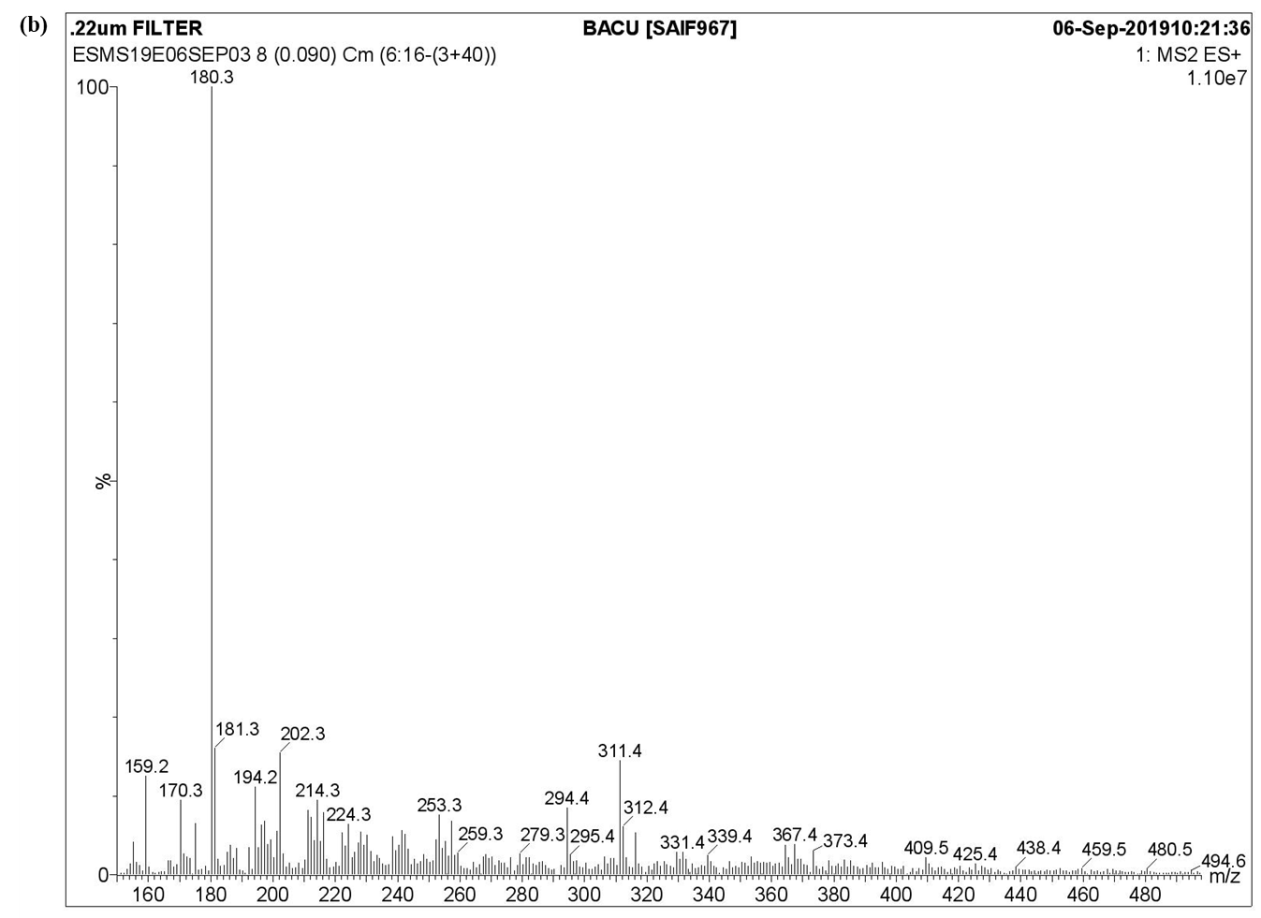
**

**
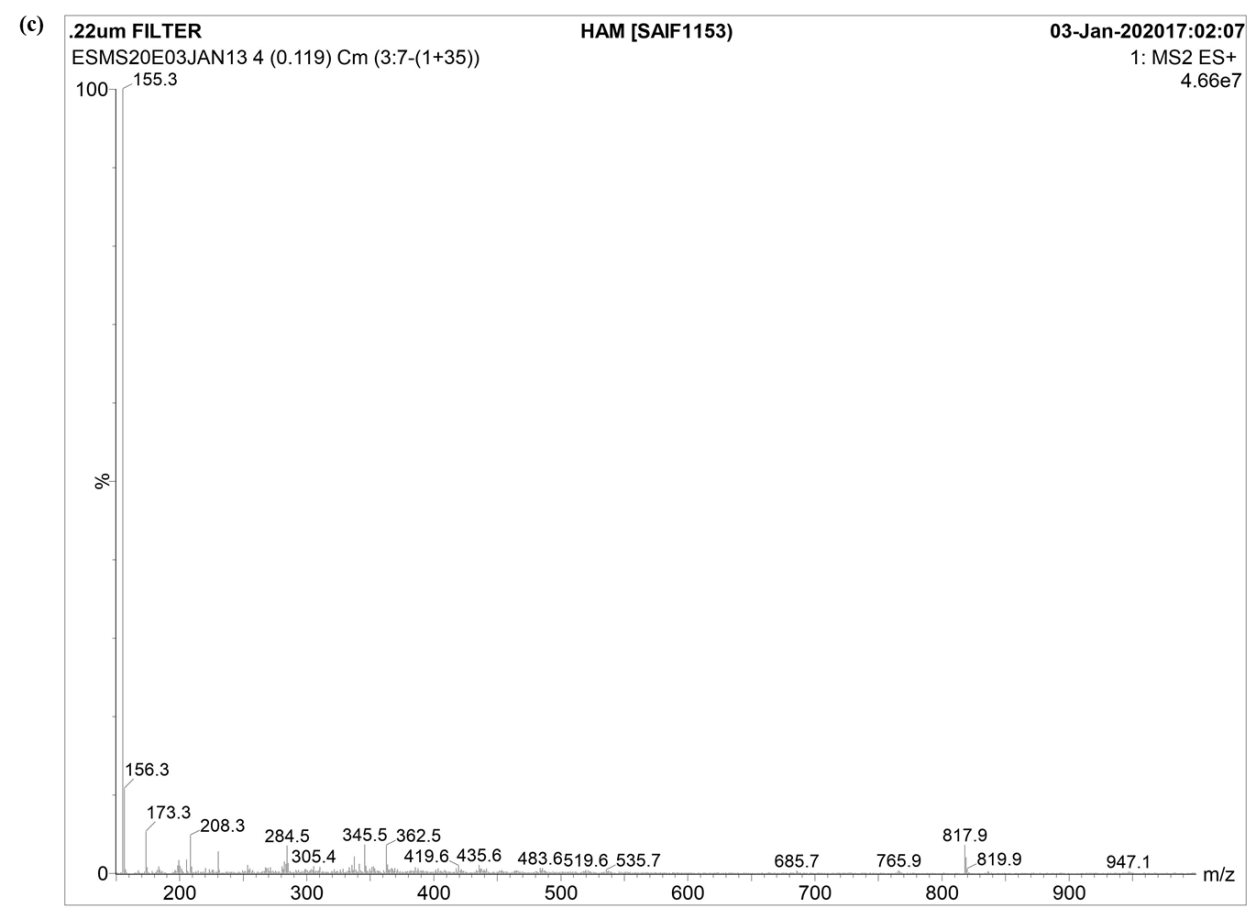
**

**
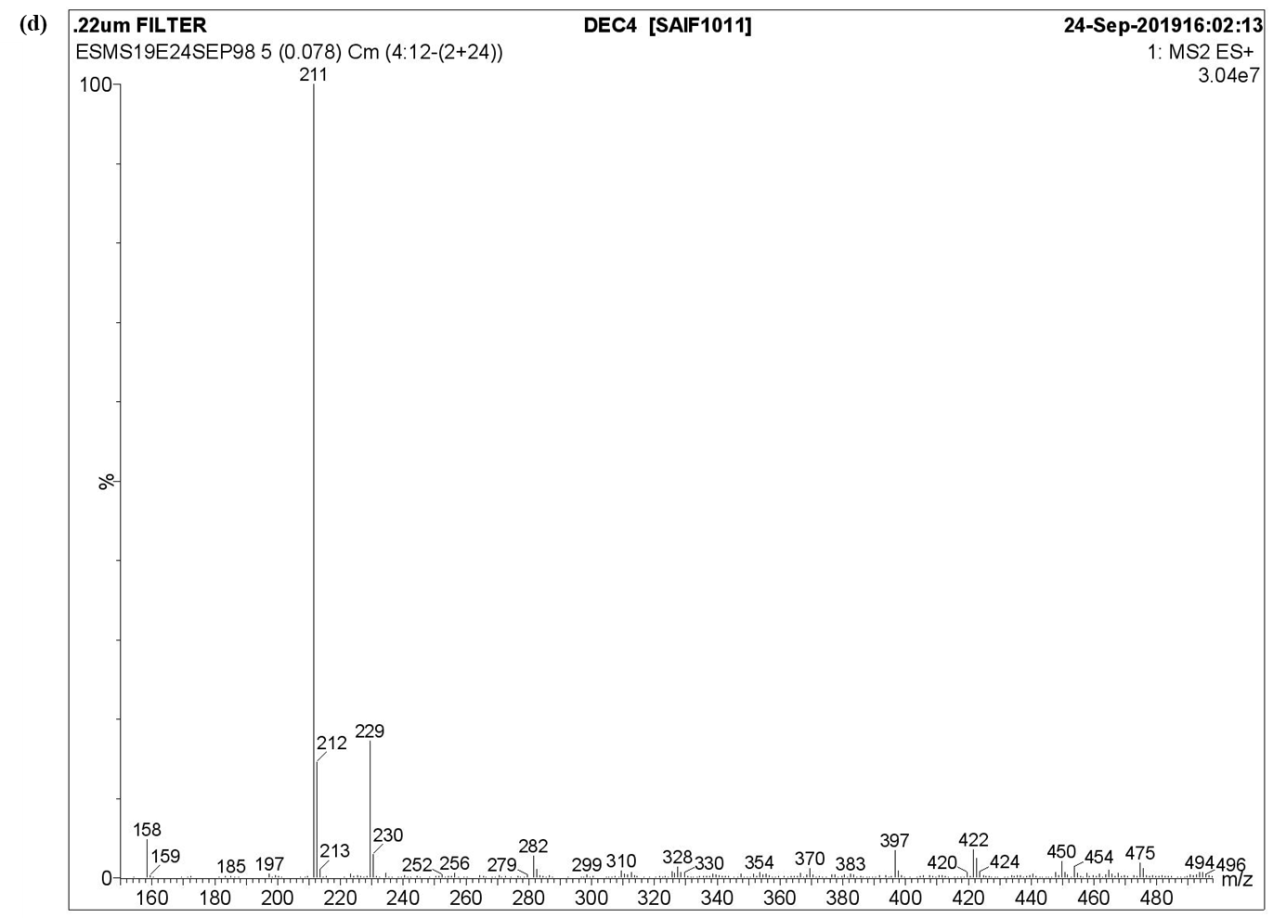
**

**
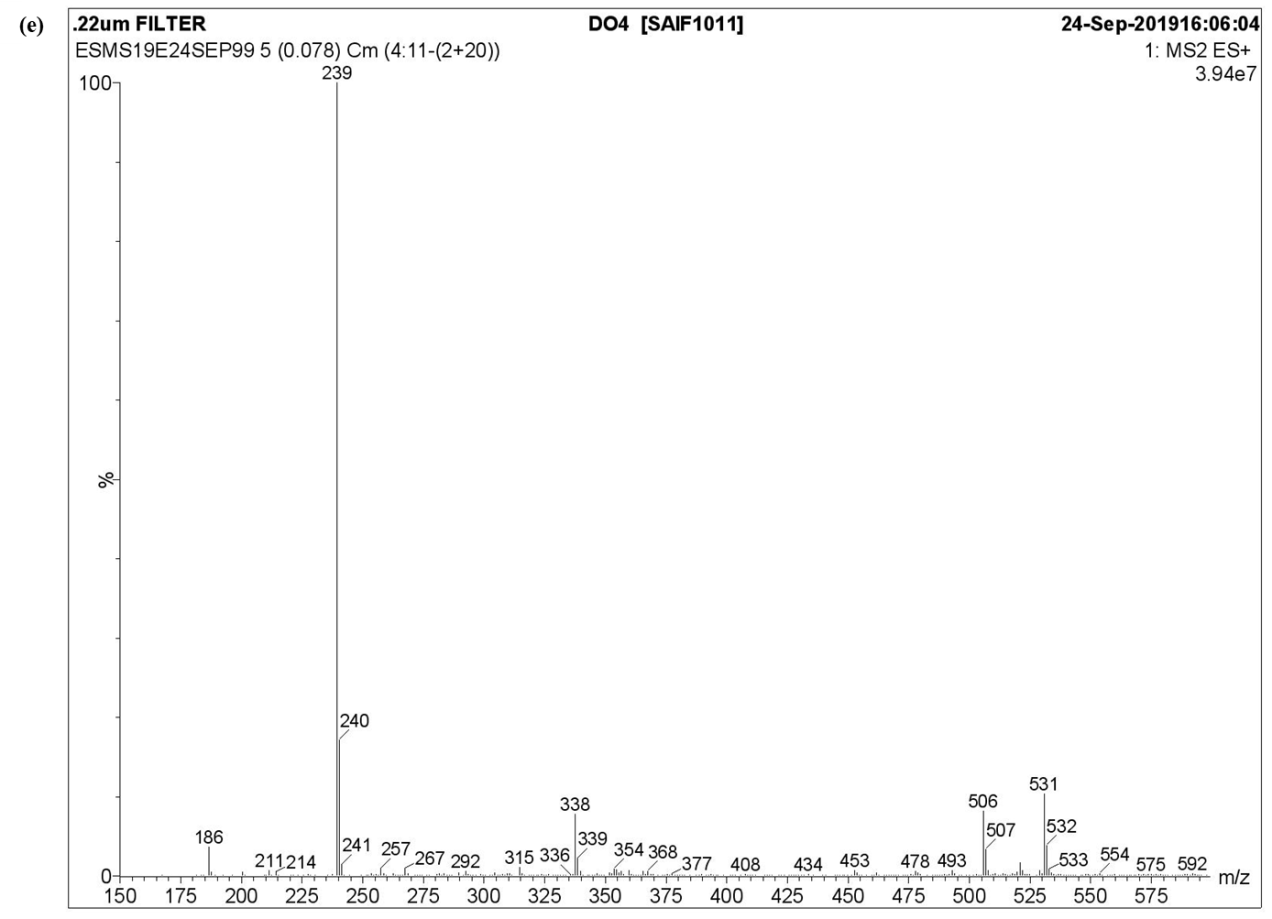
**

**Figure S3.** ESI Mass spectra of (a) n-propyliminobis-propionitrile (**N1**), (b) n-butyliminobis-propionitrile (**N2**), (c) n-hexyliminopropionitrile (**N3**), (d) n-decyliminopropionitrile (**N4**) and (e) n-dodecyliminopropionitrile (**N5**) [1].

**Antimicrobial activity**

The compounds were evaluated for their antimicrobial activities against two bacteria viz. Gram-positive bacterial strain *Bacillus subtilis* (MTCC 1305) and Gram-negative bacterial strain *Escherichia coli* (MTCC 443). Both of the strains were procured from Microbial Type Culture and Collection (MTCC), Chandigarh, India. Maintaining a temperature of 37 ºC lyophilized culture was revived in nutrient broth medium by keeping it in a rotatory shaker for 12 h. Using 0.5 McFarland standards each strain was adjusted to a concentration of 10^8^ cells/ml [2–4].

**Antibacterial Test**

The negative control (distilled water) showed no antibacterial activity while the positive control (ceftriaxone) showed the zone of inhibition against *E. coli* (18±0.1) and *B. subtilis* (17±0.3 mm) (Figure S4).


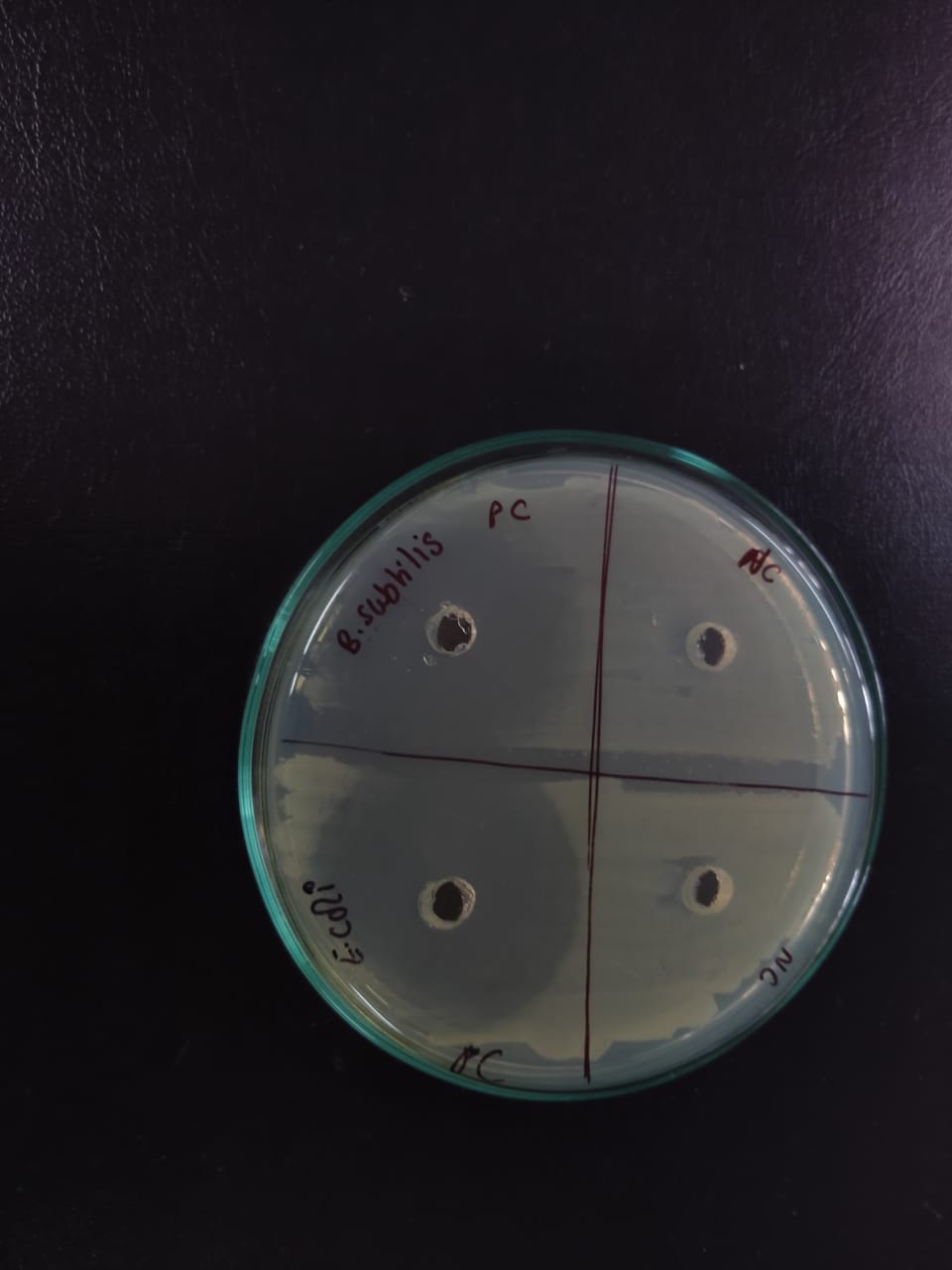


**Figure S4.** Zone of inhibition for positive and negative control against *E. coli* and *B. subtilis*

**References**

[1] P. Das, N. Devi, A. Puzari, One-pot solvent-free microwave-assisted aza-Michael addition reaction of acrylonitrile, J. Indian Chem. Soc. 99 (2022) 100411. https://doi.org/10.1016/j.jics.2022.100411.

[2] J. Mcfarland, mouths of the blanching AN INSTRUMENT FOR ESTIMATING THE NUMBER OF BAC-TERIA IN SUSPENSIONS USED FOR CALCULATING THE OPSONIC INDEX AND FOR VACCINES. Those who have used the method of Leishman or the later method of Wright for estimating the phagocytic or, J. Am. Med. Assoc. 49 (1907) 1176–1178.

[3] McFarland Standards- Principle , Preparation , Uses , Limitations Principle of McFarland Standards Preparation of McFarland Standards, (2020) 5–7. https://microbenotes.com/mcfarland-standards.

[4] D. Dutta, S. Goswami, R. Dubey, S.K. Dwivedi, A. Puzari, Antimicrobial activity of silver-coated hollow poly(methylmethacrylate) microspheres for water decontamination, Environ. Sci. Eur. 33 (2021). https://doi.org/10.1186/s12302-021-00463-5.
